# Integration of Chemoinformatics and Multi-Omics Analysis Defines ECT2 as a Potential Target for Cancer Drug Therapy

**DOI:** 10.3390/biology12040613

**Published:** 2023-04-18

**Authors:** Mohamed A. Soltan, Muhammad Alaa Eldeen, Bayan H. Sajer, Reda F. A. Abdelhameed, Fawziah A. Al-Salmi, Eman Fayad, Ibrahim Jafri, Hebatallah Emam Mohammed Ahmed, Refaat A. Eid, Hesham M. Hassan, Mubarak Al-Shraim, Amr Negm, Ahmed E. Noreldin, Khaled M. Darwish

**Affiliations:** 1 Department of Microbiology and Immunology, Faculty of Pharmacy, Sinai University, Ismailia 41611, Egypt; 2 Cell Biology, Histology & Genetics Division, Biology Department, Faculty of Science, Zagazig University, Zagazig 44519, Egypt; 3 Department of Biological Sciences, College of Science, King Abdulaziz University, Jeddah 80200, Saudi Arabia; 4 Department of Pharmacognosy, Faculty of Pharmacy, Galala University, New Galala 43713, Egypt; 5 Department of Pharmacognosy, Faculty of Pharmacy, Suez Canal University, Ismailia 41522, Egypt; 6 Biology Department, College of Sciences, Taif University, P.O. Box 11099, Taif 21944, Saudi Arabia; 7 Department of Biotechnology, College of Sciences, Taif University, P.O. Box 11099, Taif 21944, Saudi Arabia; 8 Medical Biochemistry and Molecular Biology Department, Faculty of Medicine, Benha University, Benha 13511, Egypt; 9 Pathology Department, College of Medicine, King Khalid University, P.O. Box 62529, Abha 61421, Saudi Arabia; 10 Department of Pathology, Faculty of Medicine, Assiut University, Assiut 71515, Egypt; 11 Department of Chemistry, College of Science, King Faisal University, Al-Ahsa 31982, Saudi Arabia; 12 Chemistry Department, Faculty of Science, Mansoura University, Mansoura 35516, Egypt; 13 Department of Histology and Cytology, Faculty of Veterinary Medicine, Damanhour University, Damanhour 22516, Egypt; 14 Medicinal Chemistry Department, Faculty of Pharmacy, Suez Canal University, Ismailia 41522, Egypt

**Keywords:** ECT2, pan-cancer, differential expression, prognosis, immunotherapy, drug discovery

## Abstract

**Simple Summary:**

Cancer is a leading cause of death worldwide and continuous efforts are exerted to develop novel biomarkers and therapeutic agents that could help in early diagnosis and treatment. Analysis of a pan-cancer model that includes a list of human tumors could provide us with a global solution to the heterogeneous condition of human cancers. One of the basic approaches for the detection of potential tumor biomarkers and drug targets is the assessment of specific proteins that show upregulation in cancerous tissues versus normal ones. We showed that ECT2 could be considered as a prognostic and immunological biomarker in a list of human cancers through the investigation of several databases. We also employed a chemoinformatics approach to analyze potential inhibitors for ECT2 that could finally act as antitumor agents.

**Abstract:**

Epithelial cell transforming 2 (ECT2) is a potential oncogene and a number of recent studies have correlated it with the progression of several human cancers. Despite this elevated attention for ECT2 in oncology-related reports, there is no collective study to combine and integrate the expression and oncogenic behavior of ECT2 in a panel of human cancers. The current study started with a differential expression analysis of ECT2 in cancerous versus normal tissue. Following that, the study asked for the correlation between ECT2 upregulation and tumor stage, grade, and metastasis, along with its effect on patient survival. Moreover, the methylation and phosphorylation status of ECT2 in tumor versus normal tissue was assessed, in addition to the investigation of the ECT2 effect on the immune cell infiltration in the tumor microenvironment. The current study revealed that ECT2 was upregulated as mRNA and protein levels in a list of human tumors, a feature that allowed for the increased filtration of myeloid-derived suppressor cells (MDSC) and decreased the level of natural killer T (NKT) cells, which ultimately led to a poor prognosis survival. Lastly, we screened for several drugs that could inhibit ECT2 and act as antitumor agents. Collectively, this study nominated ECT2 as a prognostic and immunological biomarker, with reported inhibitors that represent potential antitumor drugs.

## 1. Introduction

Carcinogenesis is a complex process that involves the interaction between several factors, leading to a change in the normal cell behavior and its transformation into a cancerous state [1]. The best approach to fight against cancer is to deeply analyze the various roles of the interacting components in the tumor microenvironment where this analysis could open new paths for a better understanding of this deleterious cell transformation and consequently detect potential molecular targets for early prognosis and antitumor drug discovery [2,3]. One of the basic methods to achieve that is to study the different roles of oncogenes under several forms and types of human cancers [4]. This pan-cancer analysis has witnessed a great progression in the last few years owing to the development of several databases and the suitable tools that make it possible to access and analyze a large amount of data that has been correlated regarding the differential gene expression, the prognostic value of different genes, the genetic alteration status of these oncogenes, the correlation between these gene expressions and the infiltration of several types of human immune cells, and the gene–gene interaction in the tumor microenvironment that could explain the molecular mechanism of these oncogenes [5,6]. Analysis of the available data in several cancer-related databases and integration of the findings of that analysis with the novel approaches of drug delivery such as nanomedicine-based techniques could revolutionize the field of cancer treatment and could also extend to other human diseases [7,8].

The Rho family contains a group of proteins that act as GTPases and are considered a subfamily under the Ras superfamily. These proteins perform several roles in the cell as they are responsible for organelle development, cytoskeletal dynamics, and cell movement [9]. Because of the vital roles of the Rho proteins, the mechanisms that control their signaling was extensively studied, where three groups of regulators were detected: guanine nucleotide exchange factor (GEFs), GTPase-activating proteins (GAPs), and guanine nucleotide dissociation inhibitors (GDIs) [10]. The upregulation of Rho proteins has been reported in several human cancers [11], where the mechanism of altered Rho tumorigenesis was largely studied. Firstly, Rho hyperactivity can suppress normal cellular apoptosis, leading to cell longevity. Following that, polarity loss would be observed, which introduces abnormal tumor induction. This growing mass can invade and affect the surrounding normal tissue with the help of altered adhesion proteins, which is controlled via Rho proteins; finally, the cancerous mass would be free to migrate to other tissues in the body [12].

ECT2 belongs to the family of GEFs, where its major role is the catalysis of the exchange of GDP for GTP in Rho proteins; consequently, it can control the activation status of the Rho proteins [13]. GTPases are in active status when they are connected to GTP, while their binding to GDP transfers them to the inactive condition. Because of the slow process of GDP dissociation from inactive GTPases, the binding of ECT2 to its GTPase substrates catalyzes GDP dissociation of GDP, which permits a GTP binding in its place and consequently transfer the GTPases to the active status. It is noteworthy that the binding of the new GTP molecule will lead to the release of ECT2, which in turn will activate a new GTPase. Therefore, we can collectively summarize that ECT2 can both destabilize the GTPase interaction with GDP and stabilize the nucleotide-free GTPase until a GTP molecule binds to it and, in both conditions, it is named as an activating factor for GTPases [14]. Analysis of the cellular roles of ECT2 demonstrated that it was required to signal the transduction pathways that control the process of cytokinesis [15]. Additionally, ECT2 represented an important component of the centralspindlin complex that is essential for the myosin contractile ring formation in the process of cell cycle cytokinesis. Regarding its role in tumor progression, ECT2 could associate with the PARD6A-PRKCI complex, which in turn stimulated RAC1 and finally induced cancer cell proliferation [16]. Moreover, ECT2 was found to be an activating factor for focal adhesion genes in lung adenocarcinoma, where its suppression resulted in impaired cancerous cell proliferation and adhesion [17]. In addition to that, ECT2 had the ability to promote M2 macrophage polarization in hepatocellular carcinoma, which in turn suppressed the functions of NK and T cells, which are vital immune cells for fighting against tumor progression [18]. ECT2 was also associated with the transcriptional program of cancer stem cells in gastric cancer [19] and its elevated expression was correlated with colorectal cancer progression and growth [20].

While the oncogenic roles of ECT2 have obtained a large focus in the last few years, there is still a lack of comprehensive studies that can analyze the roles of ECT2 in tumor progression from different aspects. Hence, the current study aimed to analyze the expression profile of ECT2 across a large panel of human tumors. We were also interested in studying the activation status, immune infiltration, genetic alteration, methylation condition, prognostic value, and molecular interactions of ECT2 in the tumor microenvironment to obtain an overview of the behavior of ECT2 in the tumor progression. Lastly, we tried to make screening for potential antitumor drugs through the targeting of this potential molecular target.

## 2. Materials and Methods

### 2.1. ECT2 Differential Level in Tumor and Normal Tissues

Firstly, the differential ECT2 gene expression in tumor versus normal tissue was visualized through the data deposited in the Tumor Immune Estimation Resource, version 2 (TIMER2.0) [21] and, due to the absence of normal tissue for comparison in some tumor models, we accessed the Gene Expression Profiling Interactive Analysis 2 (GEPIA2) database [22] to complete our analysis. Following that, ECT2 differential protein expression between cancerous and normal tissue was investigated through the UALCAN tool, which runs a protein expression analysis relying on the data of the Clinical Proteomic Tumor Analysis Consortium (CPTAC) [23]. Finally, we aimed to investigate the level of ECT2 in the normal, tumor, and metastatic tissue to study whether there is a correlation between the gene expression level and tumor development; for this purpose, the TNMplot web server [24] was accessed.

### 2.2. Association of ECT2 with Tumor Grade and Stage

Tumor grade demonstrates the abnormality degree of cancerous cells, while tumor stage describes the size and spreading level of the studied tumor [25]. In order to study the correlation between ECT2 expression and the tumor grade and stage, the TISIDB web server [26] was accessed. Moreover, for a comparative analysis of ECT2 association with tumor stage, we accessed the “Stage Plots” tab in the GEPIA2 database [22].

### 2.3. Survival Prognosis Analysis

We employed 2 web servers to study the correlation between ECT2 expression and patients’ survival. Firstly, the “Survival Analysis” section in the GEPIA2 database was used to obtain a heatmap describing the correlation between ECT2 expression and 2 survival modes, namely overall survival and disease-free survival. Secondly, the KM plotter [27] was utilized to investigate the role of ECT2 expression in patients’ survival in 5 different cancer models (breast, ovarian, lung, gastric, and liver cancers).

### 2.4. ECT2 Genetic Alteration and Its Correlation with Patients’ Survival

In the current stage, the cBioPortal database [28] was employed to investigate the types and effects of ECT2 genetic alterations. We started by exploring the various type of ECT2 genetic alterations in a list of human tumors. Following that, we specified the genetic mutations for deep analysis in terms of their types, sites, and implications on the survival of cancer patients.

### 2.5. Methylation and Phosphorylation Analysis of ECT2

DNA methylation is an established approach for cellular control of specific gene expression [29], where a hypomethylation status of oncogene allows for its elevated expression and finally cancer progression [30]. The current study employed 2 web servers—UALCAN [31] and SMART app [32]—to investigate the methylation status of ECT2 in tumor versus normal samples. Moving to the phosphorylation analysis, the phosphorylation cascade has been correlated with the activation status of several proteins [33] and the hyperphosphorylation status of tumor-inducing proteins has been correlated with the oncogenic activity of those proteins [34]. Here, we used the data from CPTAC to compare the phosphorylation status of ECT2 in cancerous tissues versus normal ones.

### 2.6. Assessment of ECT2 Effects on the Tumor Microenvironment

Human immune cells that infiltrate the tumor microenvironment have different roles and, sometimes, opposing outcomes [35]. Here, we accessed the TIMER2 web server to analyze the possible effect of ECT2 overexpression in controlling the immune cell infiltration in a list of human tumors. Moreover, we utilized the data from the SangerBox web server to study the correlation between ECT2 expression in cancerous tissue and microsatellite instability (MSI), tumor mutational burden (TMB), tumor neoantigens, and finally immune checkpoints.

### 2.7. Analysis of ECT2 Protein–Protein Interactions

At this stage, we aimed to analyze the network of ECT2 interacting proteins; for this purpose, we accessed the STRING database [36] under the conditions of “Experiments” as the source of interactions and “Low confidence” for the scoring. Following that, we employed the GEPIA2 database to obtain the list of the top 100 correlated genes to ECT2. Lastly, the online server (http://bioinformatics.psb.ugent.be/webtools/Venn/; (accessed on 5 January 2023)) was used to find the common genes in the above-mentioned generated lists, where the genes generated after removing duplications were submitted to the Database for Annotation, Visualization, and Integrated Discovery (DAVID) [37] to run an enrichment analysis.

### 2.8. Small Molecule–ECT2 Binding Investigation

We further investigated the molecular interaction aspects of several reported small molecules as potential inhibitors of ECT2 RhoGEF through a molecular docking-coupled explicit molecular dynamic simulation. Variable-scaffold small molecules were adopted: PubChem CID_989521 (SM1), CID_1942568 (SM2), CID_1924897 (SM3), CID_1419318 (SM4), CID_136852531 (SM5), CID_4094173 (SM6), and CID_1220023 (SM7), being previously reported as potential inhibitors of the leukemia-associated RhoGEF, namely LARG, showing the top-active molecule (SM1) with a relative Rho-LARG binding inhibition of 27.12% [38]. Binding interaction profiles and thermodynamic behavior analysis for the docked molecules at ECT2 interfaces were evaluated, as compared with the reported SM7-LARG complex. All ligands were constructed via the AutoDock Vina 12.0 software suit (Scripps Research, La Jolla, CA, USA) [39]. Structural optimization through assigning Gasteiger charges and merging non-polar hydrogens were proceeded within the software.

Atomic coordinates of the biological targets ECT2 and LARG were acquired from the respective deposited Protein Data Bank (PDB) files 6L30 [40] and 1X86 [41] and then prepared within AutoDock Vina by removing ion/solvent/water, computing Gasteiger partial charges, and introducing polar/non-polar hydrogen atoms being missed from the crystallized proteins [42]. Both *N*-terminal BRCT units were removed, keeping only the C-terminal catalytic Dbl oncoprotein and pleckstrin homology (DH-PH) domains. Further structure repairment involved correcting bond distance/angle and predicting the ionization states of polar exposed residues at pH 7.4 (0.9% *w/v* NaCl). The binding site was defined through a qualitative approach by examining concave surfaces with inherited low-thermodynamic motions (cold β-factor values) as well as high-density protein–protein interfaces between the C-terminal catalytic DH-PH domains of the guanine nucleotide exchange factors. Thus, the concave surface at ECT2, comprising Val520, Phe523, Glu524, Lys527, Glu528, Val556, Ile563, Val566, Gln567, Asp610, Arg612, Lys613, Ala616, His638, Arg639, Ser640, and Asn667, and that at LARG, including the surrounding residues of both Asn975 and Arg986 amino acids (i.e., Cys888, Ser889, Gln891, Pro892, Phe893, Glu896, Met934, Leu971, Val974, Val978, Glu982, and Gln985), met the adopted criteria as potential binding sites.

Under an assigned Vina forcefield, a docking workflow proceeded, where a Lamarckian genetic algorithm was implemented for ligand’s conformation search. Then, the genetic algorithm was applied for dock binding mode prediction. Global search exhaustiveness and maximum energy differences between binding poses were predefined at 8 and 3 Kcal∙mol^−1^, respectively [43]. Docking poses with high-scoring energies achieving significant binding contacts with reported important DH-PH domain interface residues and redocking RMSD values < 2.0 Å cut-off were selected for the best docking poses of the particular ligand. Visualizing binding interactions and docking pose analysis was performed via PyMol2.0.6 (Schrödinger, New York, NY, USA).

The best docking poses for ligand-ECT2 complexes, as well as the SM1-LARG complex, were adopted as reference structures for the conducted explicit molecular dynamics simulations using GROMACS-2019 under the CHARMM general forcefield for ligands and the CHARMM36m forcefield adopted for the target protein [44]. Compound–protein complexes were individually solvated in TIP3P cube-shaped boxes using periodic boundary conditions at a marginal distance of 10 Å [45]. Standard ionization at pH 7.4 was set for the target’s amino acids, keeping the whole system neutralized with sufficient negative and positive ions of chloride and potassium, respectively [46]. The systems proceeded through a steep descent-minimization stage for 5 ps [47], while being subsequently equilibrated under NVT (303.15 K) and then NPT (303.15 K; 1 atm. pressure) ensembles for 100 ps each [48]. Molecular dynamic runs through 100 ns proceeded under the NPT ensemble, adopting the particle mesh Ewald algorithm for computing long-range electrostatic interactions. LINCS at 2 fs integration time step size was adopted for modeling all covalent bonds [49]. The Verlet cut-off scheme was used to truncate both van der Waals and Coulomb’s non-bonded interactions at 10 Å [50]. Ligand–protein binding-free energies, across the whole simulation runs, were estimated using molecular mechanics Poisson–Boltzmann surface area (MM_PBSA) calculations, where constituting energy terms were dissected and residue-wise binding contributions were investigated [51].

## 3. Results

The abbreviations and the full names of analyzed tumors in the current study are shown in Supplementary Table S1.

### 3.1. ECT2 Elevated Expression in Several Human Tumors versus Normal Tissue

**Figure 1:**
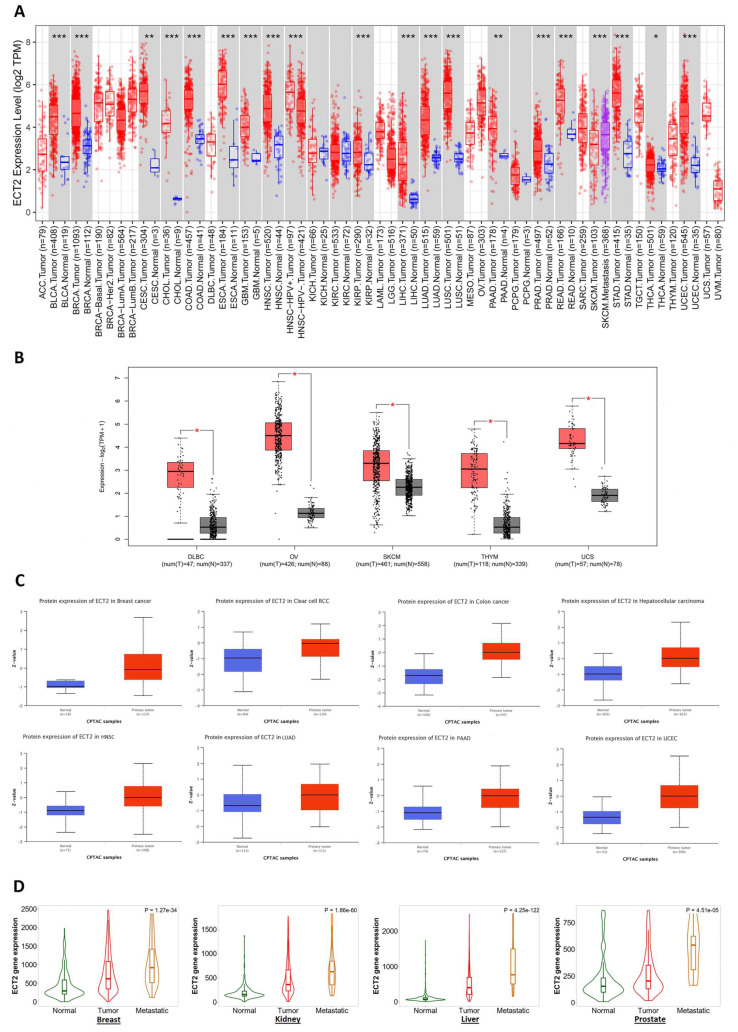
ECT2 expression assessment in human cancers. (**A**) Differential expression of ECT2 in a panel of TCGA tumors analyzed by TIMER2.0. (*: *p*-value < 0.05; **: *p*-value < 0.01; ***: *p*-value < 0.001) (**B**) Tumors experienced a significant elevation in ECT2 expression in tumor versus normal tissue when analyzed in the GEPIA2 database. (*: *p*-value with a significant score) (**C**) Tumors experienced a statistically significant higher ECT2 protein expression in the tumor sample versus normal one. (**D**) Tumors experienced a consistent positive correlation between CHD1L expression and tissue type (normal–tumor–metastatic).

TIMER2 was employed to analyze the differential expression of ECT2 between cancerous and adjacent normal tissues. Our analysis revealed that ECT2 was significantly upregulated in BLCA, BRCA, CHOL, COAD, ESCA, GBM, HNSC, KIRP, LIHC, LUAD, LUSC, PRAD, READ, STAD, UCEC (*p* < 0.001), CESC, PAAD (*p* < 0.01), and THCA (*p* < 0.05) (Figure 1A). Because of the absence of normal tissue for expression comparison in 10 tumors, we accessed the GEPIA2 database, where we found a significant elevation of ECT2 expression in 5 tumors, namely DLBC, OV, SKCM, THYM, and UCS (*p* < 0.05) (Figure 1B). On the other hand, four tumors (ACC, LGG, SRAC, and TGCT) demonstrated nonsignificant differences and only one tumor, LAML, experienced a significantly higher ECT2 expression in normal tissues versus cancerous ones (Supplementary Figure S1). Moving to the protein expression levels, eight tumors, namely breast cancer, clear cell RCC, colon cancer, hepatocellular carcinoma, HNSC, PAAD, UCEC (*p* < 0.001), and LUAD (*p* < 0.05), experienced a significantly higher ECT2 protein expression in tumor samples versus normal ones (Figure 1C), while only two tumors—ovarian cancer and glioblastoma multiforme—showed a nonsignificant difference (Supplementary Figure S2). Finally, we applied the “compare tumor, normal, and metastasis” module of the TNMplot web server to study the correlation between ECT2 mRNA levels and cancer progression and metastasis. The generated graphs (Figure 1D) illustrated that, in breast, kidney, liver, and prostate cancers, ECT2 was significantly upregulated in tumor tissues versus normal ones, a trend that was kept when we set a comparison between ECT2 expression in tumor tissues versus metastatic ones.

### 3.2. Several Human Cancers Demonstrated a Correlation between ECT2 Expression and Tumor Stage and Grade

**Figure 2:**
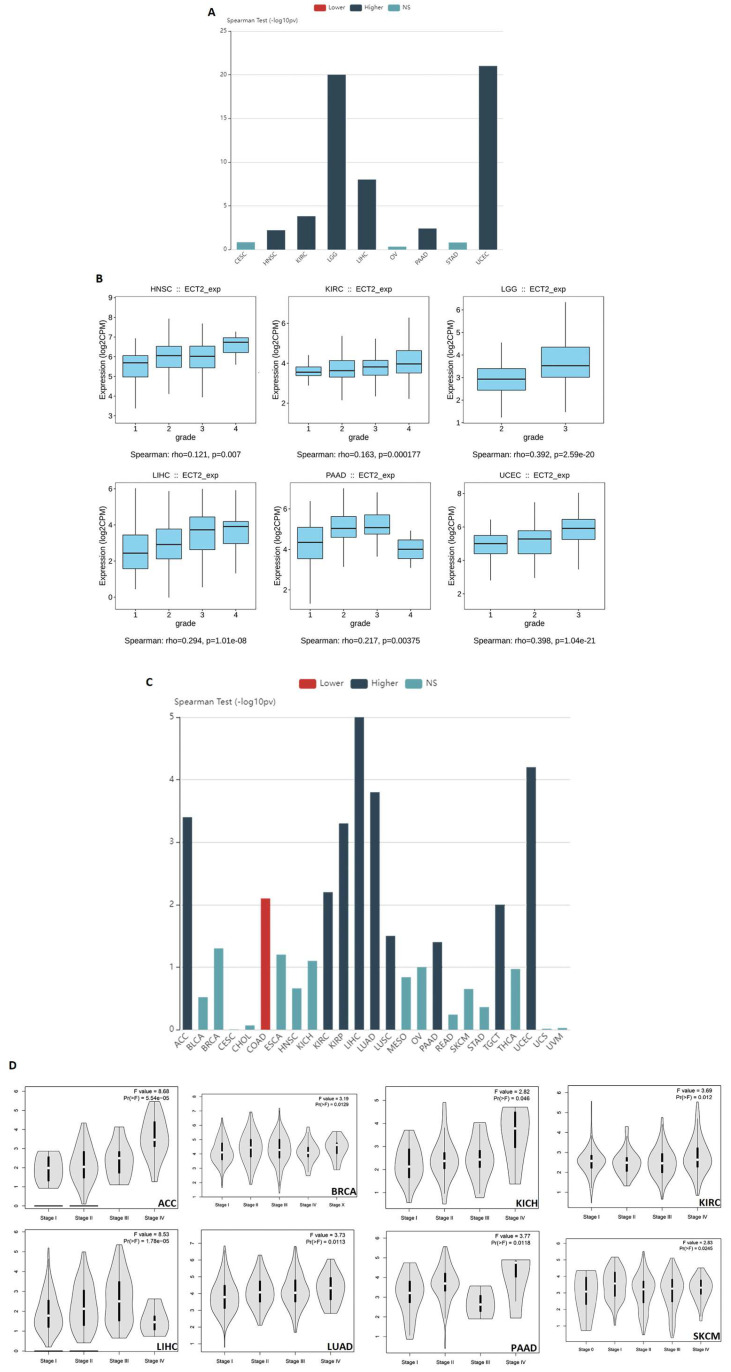
Correlation between ECT2 level and tumor stage and grade. (**A**) Bar chart showing the correlation between ECT2 level and tumor grade. (**B**) Box plot for tumors that experienced a positive correlation between ECT2 level and tumor grade. (**C**) Bar chart showing the correlation between ECT2 level and tumor stage. (**D**) Violin plot for tumors that experienced a positive correlation between ECT2 level and tumor stage.

After confirming the upregulation of ECT2 in mRNA and protein levels, we aimed to find whether this upregulation would affect the grade and the stage of human tumors. The output from the TISIDB web server demonstrated that there was a positive correlation between ECT2 expression and the tumor grade in KIRC, LGG, LIHC, UCEC (*p* < 0.001), PAAD, and HNSC (*p* < 0.01) (Figure 2A,B). Moving to the tumor stage, the results from the TISIDB web server exhibited a positive correlation with ECT2 expression in nine tumors, namely ACC, KIRC, KIRP, LIHC, LUAD, LUSC, PAAD, TGCT, and UCEC (Figure 2C), while the same assessment from the data deposited in the GEPIA2 database showed the positive correlation with ACC, BRCA, KICH, KIRC, LIHC, LUAD, PAAD, and SKCM (Figure 2D). Collectively, five tumors, namely ACC, KIRC, LIHC, LUAD, and PAAD, experienced a positive correlation between the ECT2 level and the tumor stage, based on the results from the two employed databases.

### 3.3. Increased ECT2 Levels Were Negatively Correlated with the Clinical Outcomes

**Figure 3:**
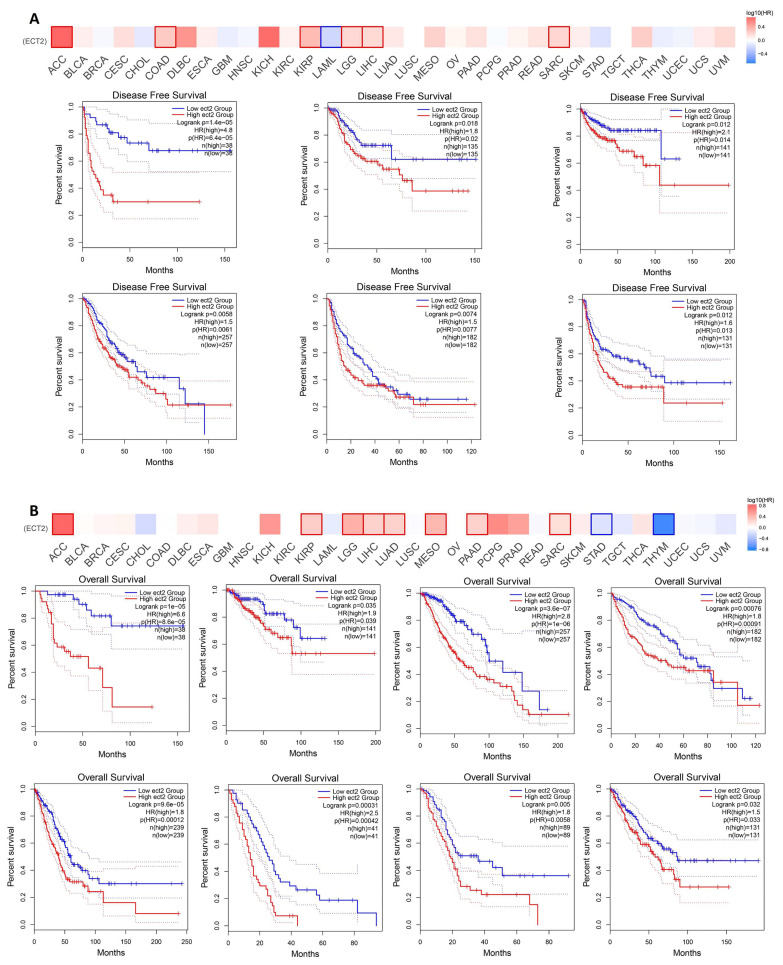
The correlation between ECT2 expression and the clinical outcome. (**A**) Disease-free survival; (**B**) overall survival as assessed from the GEPIA2 database. Boxes represent the tumors that experienced a significant correlation.

**Figure 4:**
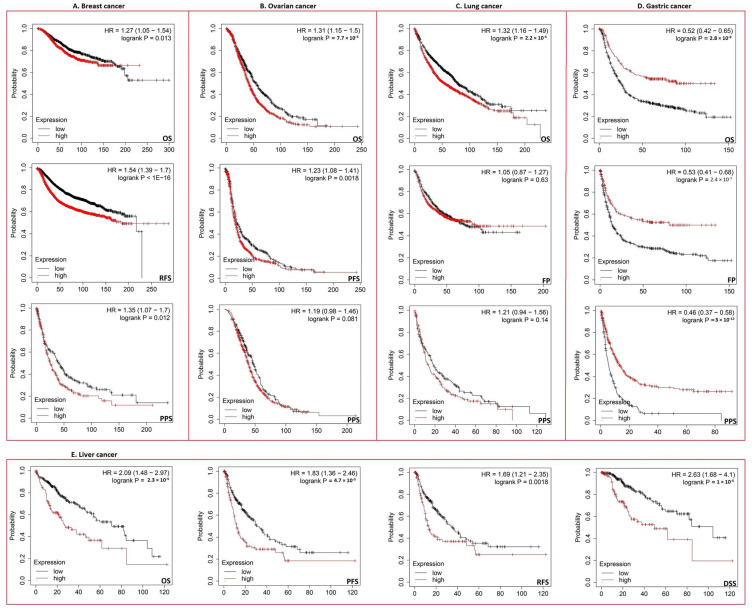
The correlation between ECT2 expression and the survival prognosis as assessed with the Kaplan–Meier plotter tool for (**A**) breast, (**B**) ovarian, (**C**) lung, (**D**) gastric, and (**E**) liver cancer.

The elevated levels of ECT2 in tumor tissues versus normal ones made us question the possible roles of ECT2 in patients’ survival; to answer that question we investigated two resources: GEPIA2 and a Kaplan–Meier (KM) plotter. Results from the GEPIA2 database revealed that six tumors, namely COAD, KIRP, SARC (*p* < 0.05), LGG, LIHC (*p* < 0.01), and ACC (*p* < 0.001), experienced a negative correlation in terms of disease-free survival (Figure 3A), where the same tumors (except COAD) also experienced a negative correlation in terms of overall survival. Not only the above-mentioned tumors but others, namely LUAD, MESO, and PAAD (Figure 3B), also showed a negative correlation in terms of overall survival. Moving to the results of the KM plotter, breast, gastric, and liver tumors demonstrated a negative correlation between ECT2 expression and patient survival in all of the analyzed models (Figure 4A,D,E), while ovarian cancer showed the same correlation in terms of overall and progress-free survival (Figure 4B). Finally, lung cancer exhibited a negative correlation only in the overall survival module (Figure 4C).

### 3.4. ECT2 Genetic Alteration Predicts Poor Patient Outcome

**Figure 5:**
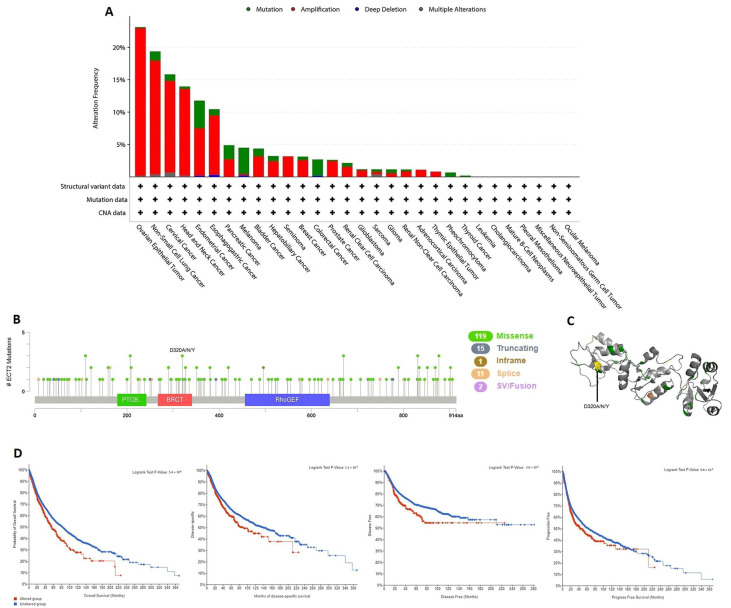
Mutation assessment for ECT2 using the cBioPortal tool. (**A**) The alteration frequency with mutation type in a panel of analyzed human cancers. (**B**) A map representation for sites and types of ECT2 mutations. (**C**) The 3D structure of ECT2, with a highlight on the most altered site. (**D**) Assessment of the correlation between ECT2 mutation and disease-free, disease-specific, progression-free, and overall survival.

The output from the cBioPortal database revealed that ovarian epithelial tumor, non-small cell lung cancer, and cervical cancer were the top three human tumors that experienced genetic alterations in ECT2, with alteration frequency approximately between 16 and 24%. Moreover, “amplification” was the dominant form of genetic alteration in most of the analyzed human tumors, except for melanoma and colorectal cancer, which exhibited “mutation” as a dominant ECT2 genetic alteration (Figure 5A). A deep analysis of the ECT2 mutation forms showed that the missense mutation was the most common form and that site D320 was one of the most altered sites in ECT2, with three reported missense mutations in uterine-related carcinoma patients (Figure 5B,C). The final interesting finding regarding ECT2 genetic alteration analysis was that, in four analyzed models, namely overall, disease-specific, disease-free, and progress-free survival, there was a negative correlation between ECT2 alterations and patient survival (Figure 5D).

### 3.5. Opposing Methylation–Phosphorylation Status of ECT2 in Several Human Cancers

**Figure 6:**
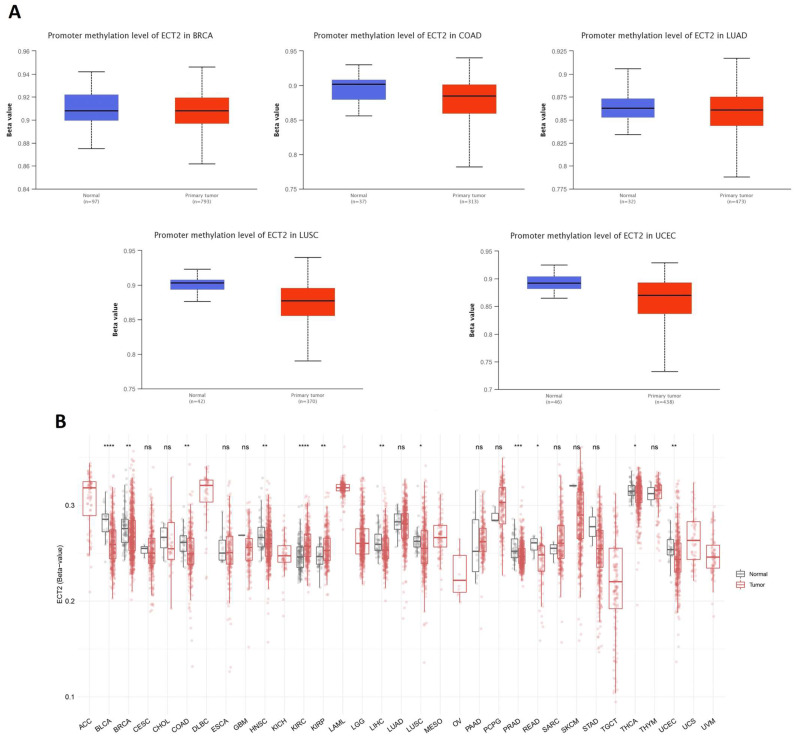
Differential methylation analysis of ECT2 in tumor samples versus normal ones. (**A**) Tumors experienced higher methylation in the ECT2 promoter region in normal samples versus tumors as assessed by UALCAN analysis. (**B**) Analysis of CpG-aggregated methylation of ECT2 in a list of human tumors. ns: *p* > 0.05; *: *p* <= 0.05; **: *p* <= 0.01; ***: *p* <= 0.001; ****: *p* <= 0.0001.

**Figure 7:**
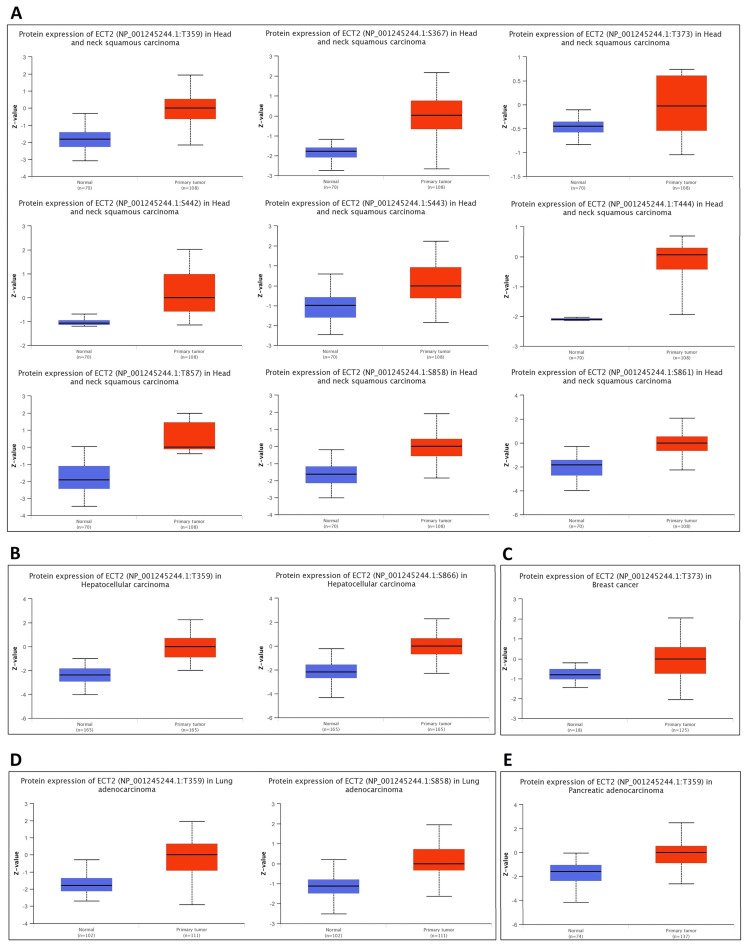
Differential phosphorylation analysis of ECT2 in tumor samples versus normal ones. (**A**) HNSC; (**B**) hepatocellular carcinoma; (**C**) breast cancer; (**D**) lung adenocarcinoma; (**E**) pancreatic adenocarcinoma.

Starting with the methylation assessment, the output of the UALCAN web server revealed that five tumors, namely COAD, LUAD, LUSC, UCEC (*p* < 0.001), and BRCA (*p* < 0.01), experienced a promoter hypomethylation status in comparison with normal samples (Figure 6A). Moreover, results of the SMART app showed that BLCA, BRCA, COAD, HNSC, LIHC, LUSC, PRAD, READ, THCA, and UCEC experienced CpG-aggregated hypomethylation compared with corresponding normal samples (Figure 6B). Moving to the phosphorylation analysis, nine positions, namely T395, S367, S443, T444, T857, S858, S861 (*p* < 0.001), T373 (*p* < 0.05), and S442 (*p* < 0.01), experienced significantly elevated phosphorylation levels of ECT2 in HNSC versus normal samples (Figure 7A). A similar pattern was observed in positions T359 and S866, when we analyzed the samples of hepatocellular carcinoma versus normal ones (Figure 7B), and position T373 in breast cancer (Figure 7C), in addition to positions T359 and S858 in lung adenocarcinoma (Figure 7D), and finally position T359 in pancreatic adenocarcinoma (Figure 7E). Collectively, ECT2 experienced a hypomethylation and phosphorylation status in tumor conditions, a property that can be correlated with its overexpression and activity in cancerous tissues versus normal ones.

### 3.6. ECT2 Expression in Cancerous Tissue Was Positively Correlated with the Infiltration of Cells with Immunosuppressive Characteristics

**Figure 8:**
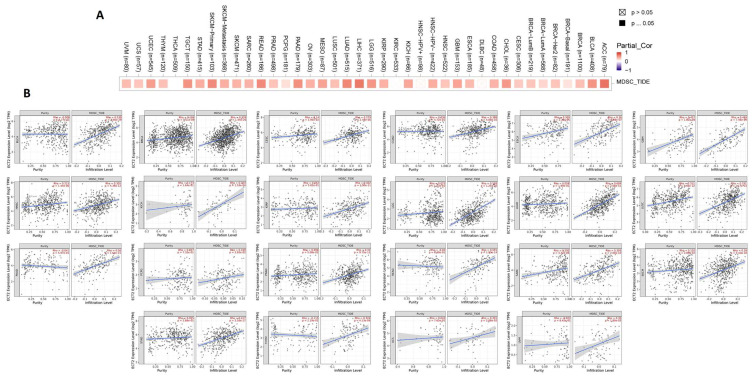
(**A**) The correlation between ECT2 expression level and infiltration of myeloid-derived suppressor cells (MDSC). (**B**) Scatter plots that demonstrate the correlation between the expression of ECT2 and the infiltration level of MDSC.

**Figure 9:**
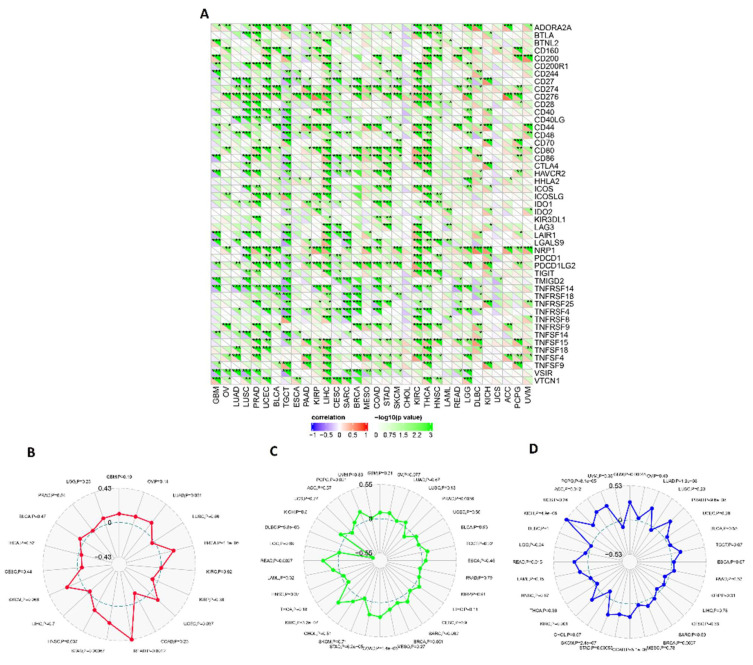
Correlations of ECT2 expression with immune checkpoints, tumor neoantigens, MSI, and TMB. (**A**) Heatmap correlating the immune checkpoints and ECT2 across a list of human tumors. (**B**–**D**) Radar charts showing the overlaps of ECT2 with tumor neoantigens, MSI, and TMB respectively. (*: *p*-value < 0.05; **: *p*-value < 0.01; ***: *p*-value < 0.001).

It was established that several types of immune cells with different roles can infiltrate the tumor. Here, we targeted two cells, namely MDSC and NKT, that have immunosuppressive and antitumor activity, respectively, to be analyzed for their potential correlation with ECT2 expression in cancerous tissue. Regarding MDSC, 90% of the analyzed tumors experienced a significantly positive correlation between ECT2 and MDSC levels (Figure 8A,B). It is important to mention that not a single tumor showed a negative correlation between ECT2 and these cells with the immunosuppressive roles. Moving to the NKT cells, the opposite correlation was demonstrated in 80% of the analyzed tumors, with the presence of only one tumor, LIHC, that showed a positive correlation between ECT2 and NKT cell levels (Supplementary Figure S3). A combined analysis revealed that BLCA, BRCA, CESC, COAD, ESCA, GBM, HNSC, KICH, LGG, LUAD, LUSC, PAAD, PCPG, PRAD, READ, SARC, SKCM, STAD, THYM, UCS, and UVM experienced a positive correlation between ECT2 and MDSC, in addition to a negative correlation between the same gene and the NKT cells. The output from the SangerBox web server showed that THCA, KIRC, and LIHC experienced a positive correlation between ECT2 and the expression of several immune checkpoints, while TGCT showed a negative correlation with most of the immune checkpoints (Figure 9A). Moreover, five tumors, namely BRCA, STAD, READ, HNSC, and UCEC, exhibited a positive correlation between ECT2 and tumor neoantigen (Figure 9B), while tumors PRAD, COAD, STAD, KIRC, READ, DLBC, and PCPG demonstrated a positive correlation between the same gene and the MSI (Figure 9C). Finally, a total number of 12 tumors, namely LUAD, PRAD, BRCA, COAD, STAD, SKCM, KIRC, READ, KICH, ACC, PCPG, and GBM, experienced a significantly positive correlation between ECT2 and the TBM (Figure 9D).

### 3.7. ECT2-Protein Interactions and Enrichment Analysis

**Figure 10:**
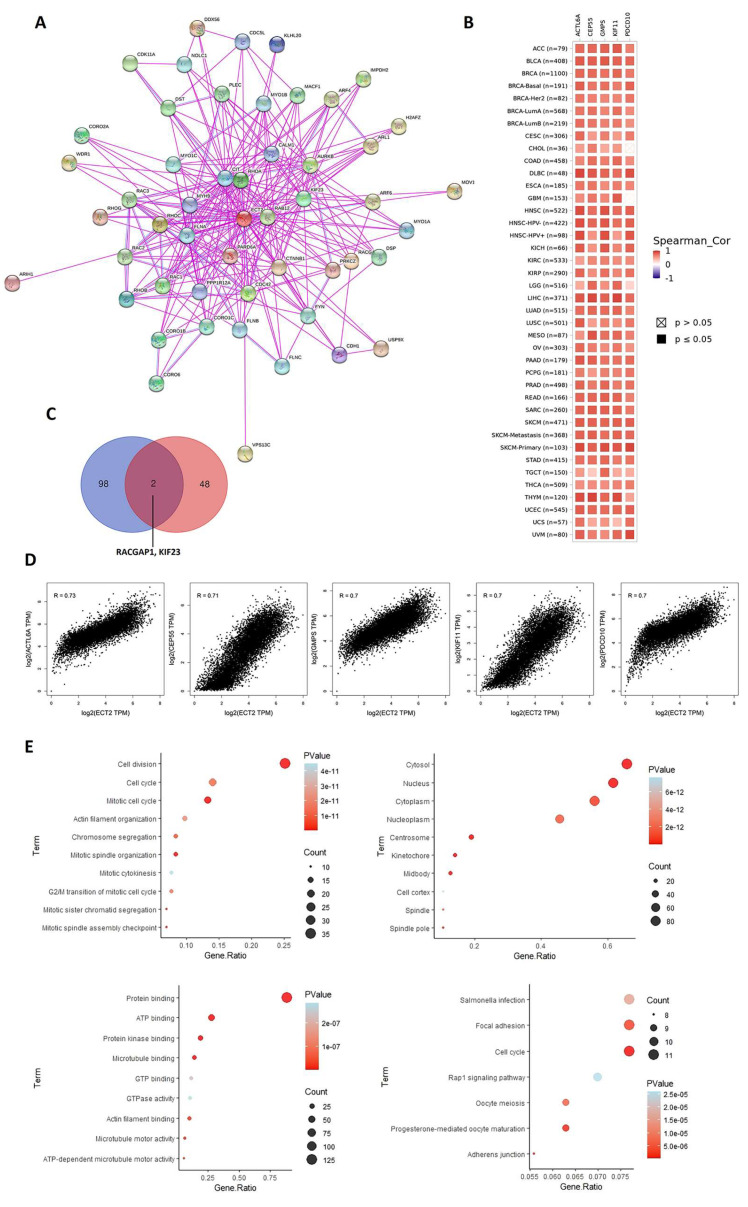
ECT2–protein network interactions. (**A**) A map of top 50 ECT2 interacting proteins as determined by the STRING database. (**B**) Heatmap for top 5 ECT2-correlated proteins in the tumor tissue. (**C**) Venn diagram showing the intersection between ECT2 interacting and correlating proteins. (**D**) Expression correlation between ECT2 and genes (ACTL6A, CEP55, GMPS, KIF11, and PDCD10) as determined by GEPIA2. (**E**) KEGG/GO enrichment analysis based on ECT2-binding and interacted genes.

Until the current stage, we have pointed out the upregulation of ECT2 in cancerous tissues versus normal samples and shown the correlation of that finding with the tumor stage, grade, patient prognosis, and immune reactivity. Therfore, it was essential to investigate the molecular mechanisms of ECT2 tumorigenic effects. First of all, 50 experimentally validated interacting proteins with ECT2 were obtained from the STRING database (Figure 10A). Following that, the top 100 ECT2 correlated proteins in the tumor microenvironment were obtained from the GEPIA2 database, where a heatmap (Figure 10B) and correlation plots (Figure 10D) for the top five correlated genes were obtained through the “Gene Corr” module under the TIMER web server and the “Correlation Analysis” tab in the GEPIA2 database. A Venn diagram demonstrating the common genes of the above-mentioned lists showed that two genes, namely RACGAP1 and KIF23, were duplicated (Figure 10C). The new gene list, derived from the combination of “ECT2-interacting genes” and “ECT2-correlated genes”, was simply created and, after duplicate removal, this new list was uploaded to the DAVID database for enrichment analysis. This gene list was enriched for cell division and cell cycle in terms of biological process, cytosol and nucleus in terms of cellular components, protein and ATP binding in terms of molecular function, and finally the KEGG pathway analysis demonstrated that these genes were enriched for cell cycle (Figure 10E).

### 3.8. Docked Small Molecules Showed Promising Binding Affinities at DH-PH Catalytic Interface

**Figure 11:**
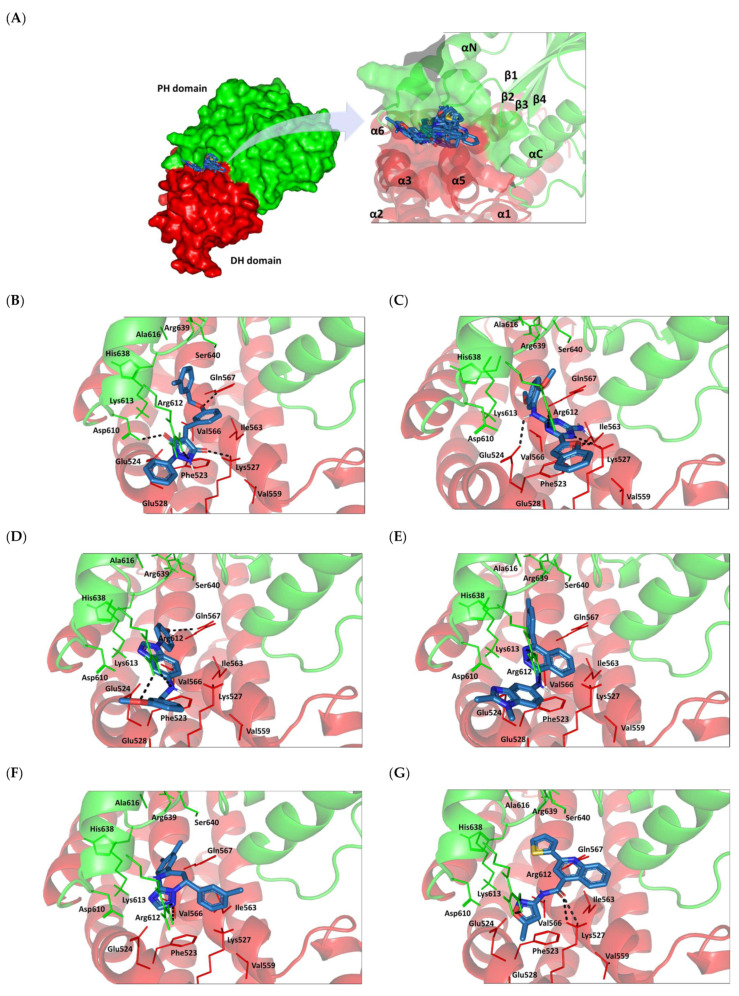

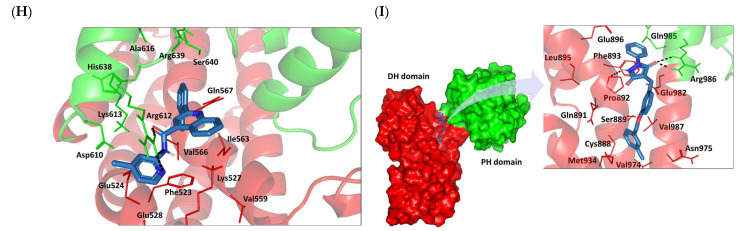
Docked binding modes and interactions for investigated small molecules at ECT2 catalytic DH-PH interface. (**A**) Overlay of docked small molecules (blue sticks) at ECT2 catalytic interface (PDB; 6L30) shown in surface representation and colored according to domain (DH and PH domains as red and green, respectively). Zoomed image defines the assigned DH-PH interface endorsed by α3, α5, α6 helices (DH domain) and αN, αC, β1–4 sheet loops (PH domain). (**B**–**H**) Predicted binding modes of the docked ligands (blue sticks); (**B**) SM1, (**C**) SM2, (**D**) SM3, (**E**) SM4, (**F**) SM5, (**G**) SM6, and (**H**) SM7 at ECT2 catalytic interface binding site. (**I**) Replicated binding mode of SM1 at LARG RhoGEF (PDB; 1X86) as reported in the literature. Residues (lines) within a radius of 5 Å from the bounded ligands are displayed, colored with regard to their position at the catalytic domains and labeled with a sequence number. Black dashed lines represent the polar ligand–target interactions.

The docked small molecules predicted excellent accommodation at the cleft interface between the DH-PH catalytic domains of ECT2 enclosed within α3, α5, and α6 helices of the DH domain, together with the αN, αC, and β1–4 sheet loops of the PH region (Figure 11A). Extended orientations/conformations were depicted for the ligands’ structural scaffold along the DH-PH interface site, having their lipopholic aryl moieties deeply anchored. Such orientation was reasoned for the dominant hydrophobic nature of the binding cleft mediated by non-polar residues, including Val520, Phe523, Val556, and Ile563 of the DH domain and Val566, Ala616, and His638 of the PH one. On the other hand, highly polar/ionized residues (Asp610, Arg612, and Lys613 of PH domain; Glu524, Lys527, and Glu528 of DH domain) were settled decorating the solvent-exposed entrance of the binding site. The depicted polar amino acids allowed favored orientations for the ligands’ hydrogen bond acceptor/donner functionalities to be preferentially directed at the solvent site.

Comparative analysis of the ligand binding modes at the ECT2 site showed preferential residue-wise binding profiles for several small molecules (Table 1). Relevant polar hydrogen bonding interactions with the solvent-exposed Arg612 sidechain were seen as consistent with all docked ligands being directed at close range towards the ligands’ polar functionalities decorating the aryl/heterocyclic scaffolds (Figure 11B–H). This ECT2 cationic residue at the PH domain even showed double polar binding interactions with some ligands, including SM3, SM4, SM5, and SM6, particularly with the benzimidazole, quinoline or purine-analogous rings. Moreover, Arg612 depicted relevant π-cationic interactions with all docked ligands, providing relevant stability for the ligands’ central and/or terminal aryl/heterocyclic rings at the ECT2 binding interface. Similar to Arg612, the polar Gln567 residue at the DH domain showed both polar hydrogen bonding and/or π-cationic interactions with different ligands, including SM1, SM3, SM4, SM5, SM6, and/or SM7. Generally, Gln567 is settled deep within the highly hydrophobic binding site, which could provide some sort of ligand selectivity towards preferential ECT2 binding. Another DH domain cationic residue, Lys527, was seen as significant for anchoring docked ligands such as SM1, SM2, and SM7 through hydrogen bonding, as well as ligands SM5 and SM7 via π-cationic interactions. Besides the depicted polar interactions, several non-polar lining residues (Val520, Phe523, Val559, Ile563, Val566, Pro570, Ile607, Ala616, His638, and/or His759) showed DH-PH interface-mediated ligand-associated hydrophobic interactions. The aromatic DH domain residue, Phe523, depicted π-π or even π-hydrogen bonding, with aromatic functionalities of almost all docked ligands. On the other hand, the sidechain carbons of Glu524 and Lys613 were found in close proximity to several ligands hydrophobic skeletons, mediating close-range van der Waal interactions. Combining both polar and hydrophobic interactions provided both distinct and variable extent residue-wise binding profiles for each ligand being correlated to favorable docking scores, with binding energies ranging from −5.469 to −6.369 Kcal.mol^−1^. Ligands such as SM2, SM3, and SM4 were assigned the highest docking scores, being associated with the extended and diverse nature of binding interactions towards ECT2.

The adopted docking protocol was validated via a triple approach, where obtaining low RMSD values (<2.0 Å) between docked and redocked poses conferred the validity of the adopted docking procedure and the furnished docking modes and energies were of ensured biological significance [52,53,54]. Secondly, the docking of SM1 at the LARG DH-PH domain interface was replicated, as previously reported by Shang et al. [38], in a way to partially ensure the adequacy of the applied docking protocol. Interestingly, SM1 showed comparable extended anchoring at the LARG catalytic domain, being stabilized through polar hydrogen bonding with Pro892 and Arg986 via its pyrazolidindione central scaffold (Figure 11I). Dominant hydrophobic interactions with Phe893 (π-hydrogen), van der Waal side chain of Glu896, Asn975, and Gln985, as well as non-polar contact with Cys888, Phe892, Leu895, Met934, Leu937, Leu971, Val974, and Val978 lining residues, were correlated with the LARG’s interface hydrophobic nature. Finally, validation of the obtained ligand-ECT2 complexes also proceeded through subsequent explicit molecular dynamic simulations using these docked poses as starting structures while comparing them with the reference-simulated SM1-LARG system.

### 3.9. Several Small Molecules Exhibited Thermodynamic Stability at DH-PH Catalytic Interface

**Figure 12:**
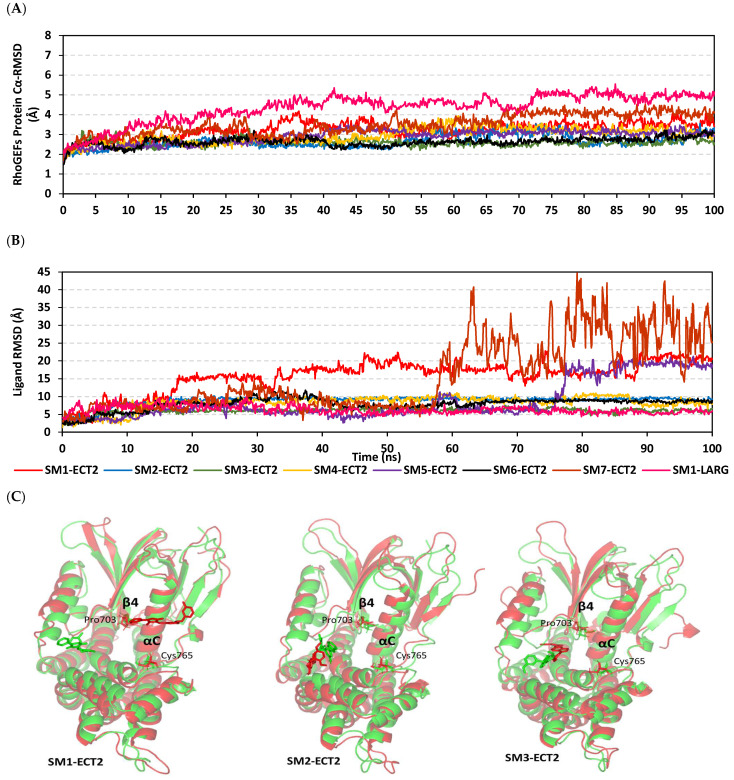

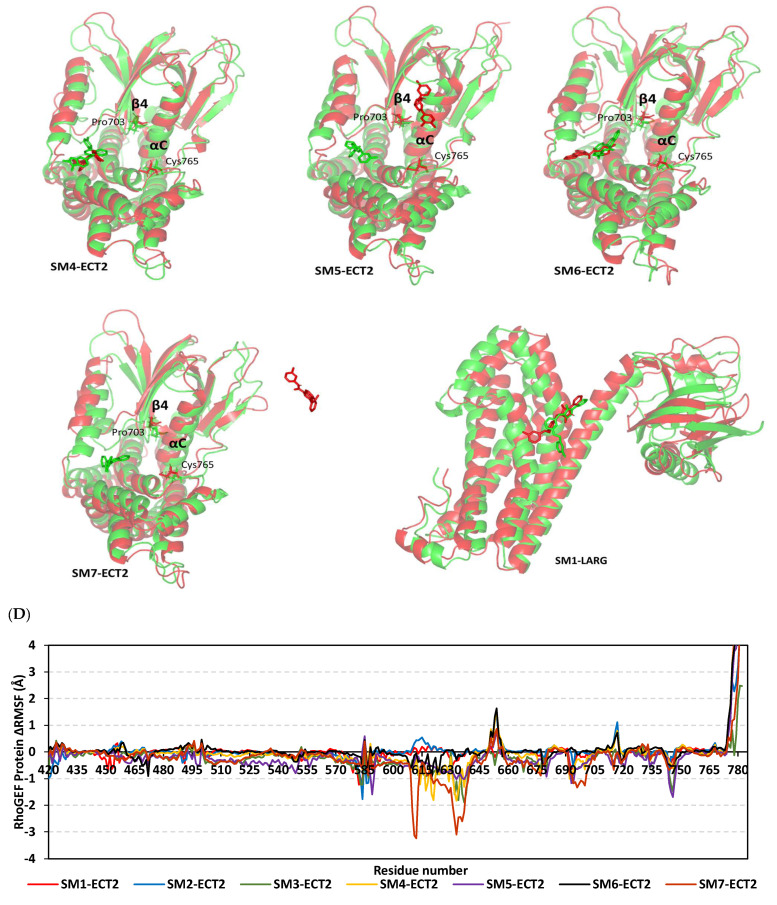
Thermodynamic stability of the simulated small molecules bound to RhoGEFs. (**A**) Alpha-carbon atom RMSDs for RhoGEF proteins. (**B**) Sole ligand RMSDs in relation to simulation time frames in nanoseconds (ns). (**C**) Overlaid ligand/RhoGEF trajectories at initial and final time frames. Ligands (sticks) and bound RhoGEF proteins (cartoon) are colored green and red with respect to 0 ns and 100 ns extracted frames. For ECT2 proteins, Pro703 β4-sheet tip and C-terminal Cys765 at the PH domain are annotated. (**D**) Difference RMSF (ΔRMSF) trajectories for ECT2 target proteins along the whole molecular dynamic simulations in relation to apo/unliganded state.

Typical thermodynamic behaviors were depicted for the simulated proteins since carbon-alpha RMSDs showed elevation across initial times owing to system relaxation followed by leveled-off trajectories around respective averages for more than half of the simulation runs. In reference to corresponding initial structures, the root-mean standard deviation (RMSD) trajectories were monitored for each of the simulated RhoGEF target protein and bound ligand molecules to investigate their conformational changes and relative stabilities [55]. Interestingly, the monitored RMSDs for all ligand-bound RhoGEF proteins were at lower average values and less fluctuating trajectories compared with the apo/unliganded ones (2.91 ± 0.34 Å versus 3.40 ± 0.34 Å for ECT2s and 4.08 ± 0.78 Å versus 4.35 ± 0.71 Å for LARG models) (Figure 12A). The latter apo versus holo dynamic behavior conferred the compactness and gain of stability for the complexed target proteins upon ligand binding. Notably, the LARG model depicted higher RMSD values compared with those of the simulated ECT2 ones. Comparative RMSDs for bound ECT2 proteins showed greater stability and minimal fluctuations for most simulated bound proteins, yet only for those in complexes with SM1 and SM7 were slightly higher RMSD tones depicted (3.22 ± 0.40 Å and 3.49 ± 0.52 Å, respectively). Despite differential ECT2 protein RMSD tones across the simulation window, all bound ECT2 proteins managed to converge around a mean RMSD of almost 3.19 Å at the end of the simulation runs (100 ns). The latter thermodynamic behavior is considered adequate with relevant protein stability and convergence, and confers molecular dynamic validity with no need for further time extensions.

Moving toward the sole ligand RMSDs, it was obvious that almost all ligands were confined and stable at the bound target-binding site pocket, as illustrated in Figure 12B. Across the simulation runs, limited fluctuations and steady trajectories were assigned for SM2, SM3, SM4, and SM6 in complexes with ECT2. None of the latter simulated ligands exceeded 10 Å, while the ligand RMSD tones were just below three-fold the RMSD trajectories of their bound proteins. The latter data conferred system convergence and great ligand stability at the reference ECT2–DH-PH interface [56,57]. On the contrary, the pyrazolidindione-based ligand, SM1, showed an abrupt increase in its RMSDs beyond 15 ns, where the upraised tones were then maintained along the RMSD plateau (17.59 ± 2.20 Å) until the end of the simulation run. Similar abrupt RMSD tone increases were seen with the quinoline ligand, SM5, yet at a much delayed time of the simulation run being not prior to 75 ns. Interestingly, both comparable upraised RMSDs for SM1 and SM5 indicated a significant ligand orientation shift from the initial DH-PH binding site in order to be then maintained at a new surface pocket that could be opened during the simulation run. This could partially explain the higher RMSDs for SM1- and SM7-bound proteins. Finally, only SM7 showed the most fluctuating RMSDs beyond half of the molecular dynamics simulation run, indicating significant ligand drift towards the solvent side far from the ECT2 interface. Unlike the orientation drift seen for SM1 at ECT2, the same ligand showed the steadiest ligand RMSDs (6.29 ± 1.06 Å) at the LARG RhoGEF–DH-PH interface, conferring relevant ligand-pocket stability.

The time evolution of ligand–target complex conformations and ligand orientation was monitored via the overlaid timeframes at the beginning and end of the simulation runs (Figure 12C). Limited orientation changes were illustrated for the simulated SM2, SM3, SM4, SM5, and SM6 bound to ECT2, which was consistent with their corresponding RMSDs. However, some ligands, including SM2, SM3, and SM6, showed deeper orientation at the DH-PH interface pocket at the end of the dynamic runs. Regarding SM1 and SM5, the ones with the upraised RMSD tones, they depicted major drift from the initial DH-PH site while settled at the PH interface close to the groove endorsed by αC-helix and β-sheets. Interestingly, both latter ligands that anchored at close range from C-terminal PH-domain residues, Pro703 and Cys765, have been reported as important for ECT2 autoinhibition [40]. On the contrary, SM1 depicted a major drift far away from the DH-PH site and towards the solvent side. Interestingly, all ECT2-bound ligands, even the two that drifted towards the PH side, maintained the PH domain sequestration of the DH catalytic interface where the Pro703 at β4-sheet tip and C-terminal Cys765 of the PH domain were anchored at separate DH grooves. Such PH-associated ternary structure insertions were more disordered in the case of the simulated SM7-ECT2 model compared with the others, particularly as SM7 drifted away towards the solvent side. Notably, SM1 showed limited conformational/orientation shift at the LARG interface, except for a twist for its terminal methyl benzyl ring owing to rotation around its dihedral angle.

Monitoring the RMS_Fluctuations (RMSF) of the bound and apo target proteins in relation to their alpha-carbon references provided further stability analysis by dissecting the proteins’ flexibility/immobility profiles down to their constituting residues [58]. We adopted normalized RMSF data across the simulated models, where difference RMSF (ΔRMSF) trajectories were estimated for each ligand-bound RhoGEF protein in relation to unliganded/apo target state (ΔRMSF = RMSF^apo^ − RMSF^holo^) [52]. Reported significant ternary structural mobility/flexibility was assigned for residues with much higher negative ΔRMSFs below a cut-off of 0.3 Å [47,52]. Similar to findings with RMSD analysis, higher flexibility and mobility tones across almost all protein regions were assigned for the apo ECT2 target protein in relation to its different holo states. The latter was obvious, since positive ΔRMSF values (high stability) were depicted for most of the protein regions (Figure 12D). The latter dynamic behavior confers the significant positive impact of ligand binding on the stability of ECT2 and such influence was extended beyond the canonical catalytic site, affecting even the far target regions. As expected, the DH domain inherited many more stability patterns compared with the PH-domain regions, owing to the latter ternary structure comprising several β-loops interconnecting the eight β-sheets and just two α-helices. One of the most inherited flexible PH regions was the residue range across Cys630-to-Val645 at all simulated models (ΔRMSF up to −3.24 Å with SM7), which actually represented the distorted loop connecting the αN-helix with the β1-sheet. Other PH-domain β-loops were flexibly assigned, including Lys675-to-Leu696 and Met742-to-Glu750 being reported with low secondary structure compactness and intermolecular bindings [59]. On the contrary, the peak stable anti-parallel β-sheets (Val642-to-Glu652) were assigned relevant immobility profiles (ΔRMSF up to 1.63 Å). Notably, the ECT2 C-terminal region at the PH domain was assigned a great immobility profile compared with the amino terminus presented at the DH region. The stable C-terminus included the αC-helix tip anchored at the DH groove, which confers stable autoinhibition of the catalytic DH domain via the PH region. Similar stability findings were assigned for β4-sheet Pro703 and vicinal residues endorsing the coverage of the DH catalytic interface.

### 3.10. Hydrophobic Potentials Dominated the Binding-Free Energy Contributions at DH-PH Catalytic Site

**Figure 13:**
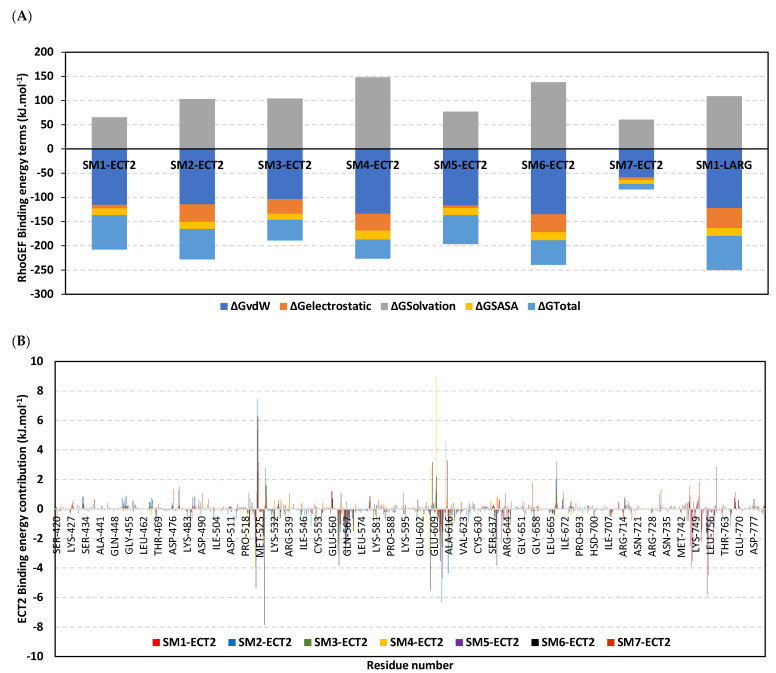
Free binding energies for ligand/ECT2 complexes based on MM_PBSA calculations. (**A**) Total free binding energies and their constituting energy terms. (**B**) Residue-based energy contributions within the free binding energies.

Free binding energy calculations via the trajectory-oriented MM_PBSA approach were performed for understanding the nature of ligand/RhoGEF binding, estimating affinity magnitude, as well as individual energy contributions of the key binding residues [60]. Typically, MM_PBSA is reported with comparable accuracy in relation to free-energy perturbations yet with lower computational expenders [61]. Interestingly, van der Waal hydrophobic potential energy contributions (ΔG_vdW_) dominated the free-binding energies of all simulated ECT2 models (Table 2 and Figure 13A). Hydrophobic contributions were more than three-fold higher than those of Coulomb’s electrostatic potentials (ΔG_electrostatic_). Similarly, the van der Waal domination over the electrostatic potential energies was also seen with the SM1-LARG system, conferring the comparable nature of the DH-PH interface at both RhoGEFs. Notably, ligands that managed to stay confined at the DH-PH interface site were assigned with higher electrostatic potentials than those being drifted towards the PH-domain side (SM1 and SM5) or even the one that drifted far away to the solvent side (SM7). Nevertheless, those that maintained their grip at the ECT2 interface (SM1 and SM5) managed to partially compensate their poor electrostatic potentials through van der Waal hydrophobic contacts. Owing to the higher hydrophobic characteristics of SM1 being inherited within its exetended aromatic/heterocyclic architecture, the ligand overcompensated the electrostatic loss more than SM5, which was correlated with higher total binding energy (ΔG_total_) for SM1. Another interesting observation was that higher electrostatic potentials were associated with higher positive values of the repulsive polar solvation energies (ΔG_Solvation_), which would compromise the ΔG_total_ for several site-oriented ligands; this was obvious when comparing SM1 against SM2, SM3, SM4, and SM6 at ECT2 or even against SM1 at the LARG interface, where all the latter ligands came second to the SM1-ECT2 model. Regarding the solvent-drifted ligand, SM7, the quinoline-based molecule achieved the worst free binding energies, as, being far from the ECT2 site for half the simulation run, it failed to compensate for such binding energy loss. Finally, evaluating the total non-polar interactions (summation of apolar solvation; ΔG_Solvent-accessible surface area/SASA_ and ΔG_van der Waal_) [54,62] of both ECT2 versus LARG systems (−128.96 versus − 137.88 kJ.mol^−1^) illustrated that the LARG interface is bigger and more hydrophobic in nature and is suitable for the accommodation of large-sized non-polar molecules [40,59].

Dissecting the total free binding energies down to residue-wise levels has provided more insights concerning key ligand–residue interactions relevant to system stability. As illustrated in Figure 13, several residues illustrated significant contributions within the calculated free binding energy. Most recognized binding residues were those initially depicted at the preliminary docking study, including Phe523 (−0.76 to −5.32 kJ.mol^−1^), Lys527 (−1.83 to −7.83 kJ.mol^−1^), Ile563 (−0.78 to −3.11 kJ.mol^−1^), Arg564 (−0.29 to −1.57 kJ.mol^−1^), Val566 (−0.96 to −3.10 kJ.mol^−1^), Gln567 (−0.89 to −2.45 kJ.mol^−1^), Arg612 (−1.56 to −3.52 kJ.mol^−1^), Arg613 (−1.21 to −6.35 kJ.mol^−1^), and Ala616 (−0.24 to −4.27 kJ.mol^−1^). As expected, ligands confined at the DH-PH interface showed higher negative residue-wise contributions at both DH and PH interface residues than those that drifted away. However, several αC-helical residues were particularly significant for energy contributions at SM1 and SM5 systems, including Leu747, Pro748, Trp752, Met755, and Arg758, reaching up to −5.83 kJ.mol^−1^ binding energy. The deep interface residue Arg639 was more significant for SM2 and SM6 binding compared with any other ligand. On the other hand, several polar/ionized residues such as Glu524, Glu528, and Glu560 contributed negatively to total binding energy, with high positive repulsive energy values. Regarding the SM1-LARG system, several non-polar residues contributed well within the free binding energies, including Cys888 (−1.14 kJ.mol^−1^), Pro892 (−4.79 kJ.mol^−1^), Leu (−2.19 kJ.mol^−1^), Met934 (−5.57 kJ.mol^−1^), and Val978 (−5.75 kJ.mol^−1^), while polar Arg986 contributed in electrostatic potentials (−6.39 kJ.mol^−1^). On the contrary, polar residues such as Gln891 2.46 kJ.mol^−1^, Glu896 2.8 kJ.mol^−1^, and Glu982 12.24 kJ.mol^−1^ compromised the SM1-LARg stability.

## 4. Discussion

Rho GTPases are molecular switches with a main function of signal transduction, therefore they control the basic cellular processes, including cytoskeleton organization, cell migration, proliferation, and survival, where nucleotide-exchange factors (GEFs), to whom ECT2 belongs, regulate the activity of these molecular switches [63]. Consequently, ECT2 has a vital role in controlling cell proliferation, division, survival, and apoptosis [64]. Moreover, ECT2 has been reported to be overexpressed in a panel of human cancers [65]. In ovarian cancer, ECT2 was reported to stimulate cellular transformation by acting as a RhoGEF specifically within the nucleus [66]. Regarding breast cancer, analysis of 165 breast cancer specimens and 100 normal samples nominated ECT2 as one of the main causes of the occurrence and development of that cancer [67]; in addition to that, a recent study reported that an increased ECT2 level was highly associated with advanced TNM stage [68]. Moving to gastric cancer, an analysis of 52 cancerous specimens attributed ECT2 to the progression of gastric cancer [69]; another study investigated the ECT2 expression gene in tissues and serum of gastric cancer patients and reported it as a new diagnostic marker [70]. Moreover, ECT2 was detected by RT-PCR and was found to be overexpressed in pancreatic tumor tissues [71]. Colorectal cancer is another form of human cancer where upregulation in ECT2 expression predicts an unfavorable prognosis [72]. Another report that studied the same tumor recommended the utilization of ECT2 expression as a sensitive biomarker for the diagnosis and monitoring of the patients [73]. Furthermore, ECT2 overexpression was reported to stimulate the polarization of tumor-associated macrophages in hepatocellular carcinoma, a cell that suppress the functions of NK and T cells in the tumor microenvironment [18]. The same tumor, hepatocellular carcinoma, was promoted for recurrence also by the action of ECT2 [74] and, along with NEK2 and DLGAP5, ECT2 was identified via a genome-scale analysis to act as a prognostic biomarker in lung cancer [75].

While the oncogenic properties of ECT2 have been discussed in many reports, there is a lack of a comprehensive studies that analyze the behavior, molecular interactions, and effects of this oncogene in a panel of human tumors. It is well established that the tumor microenvironment is a complex system and it is important to deeply study and analyze the basic molecular interactions in that environment to put our hands on potential diagnostic and therapeutic pathways [6,76]. The current study started with a differential analysis, where we confirmed the reported overexpression of ECT2 in cancerous tissues versus normal ones. Then, we moved to correlate this overexpression with tumor grade and stage; the combined analysis showed that four human tumors, namely KIRC, LIHC, UCEC, and PAAD, experienced a positive correlation between ECT2 overexpression and both tumor grade and stage. Survival analysis is an essential approach for investigating the clinical outcome as a result of a specific point of analysis, such as therapeutic intervention or targeted gene expression [77], and, due to its importance, we analyzed the correlation between ECT2 overexpression and the patients’ survival, where several human tumors, including COAD, KIRP, SARC, LGG, LIHC, and ACC, experienced a negative correlation between ECT2 and the clinical outcome in terms of overall and disease-free survival. Moreover, the mutation status of the gene has been largely correlated with patients’ survival. For example, *KRAS* mutations were correlated with a poor clinical outcome in pancreatic [78] and lung cancer patients [79], *TP53* mutation predicted poor survival in lung cancer patients [80], and STK11 mutations were negatively correlated with patients’ survival with NSCLC [81,82]. Consistent with these reports, the current study found a negative correlation between ECT2 mutation and patients’ survival in terms of overall, disease-specific, disease-free, and progress-free survival.

Gene methylation status is a basic cellular technique for controlling gene expression [83,84], where, under the tumor condition, tumor suppressor genes are generally hypermethylated (silenced) [85], while oncogenes are hypomethylated (activated) [86]. Consistent with that, the current study revealed the hypomethylation status of ECT2 (as both promoter and CpG-aggregated hypomethylation) in a large set of human cancers. An opposed status was observed when we considered the phosphorylation status of ECT2 in cancerous tissues versus normal ones as several positions, including T395, S367, S443, T444, T857, S858, S861, T373, S442, T359, and S866, demonstrated hyperphosphorylation in several human cancers. Again, this observation matchedwith the essential role of oncogene phosphorylation to induce tumor progression; examples include the phosphorylation of BRD4 [87], Smad3 [88], and EGFR [89].

Reinvigoration of malfunctioning immune cells to fight against a growing tumor opened the door for a new branch of “tumor immunotherapy” that has expanded largely in the last few years [90]. From this point, we aimed to correlate the expression of ECT2 in the tumor microenvironment with the infiltration of immune cells. The analysis of MDSC infiltration, which is known for immunosuppressive roles [91], showed a positive correlation with ECT2 in most of the analyzed human tumors. This point is of great importance, as MDSC inhibition was correlated with the improved response to immune checkpoint inhibitors (ICI) [92], therefore the combination of ICI with ECT2 inhibitors is an antitumor approach with a potential synergistic effect. In addition to its effect on MDSC infiltration, the current study found that ECT2 was positively correlated with the infiltration of NKT cells, which are known for their antitumor activity [93]. Collectively, 21 tumors, namely BLCA, BRCA, CESC, COAD, ESCA, GBM, HNSC, KICH, LGG, LUAD, LUSC, PAAD, PCPG, PRAD, READ, SARC, SKCM, STAD, THYM, UCS, and UVM, experienced a positive correlation between ECT2 and MDSC, in addition to a negative correlation between the same gene and NKT cell infiltration, which potentiates the selection of ECT2 as a therapeutic target for enhancing the immune response against human tumors. As ECT2 showed important roles in affecting the clinical outcome, tumor stage, grade, and immune cell infiltration, we investigated the molecular interactions of ECT2, where two proteins, namely RACGAP1 and KIF23, were common ones in the two lists of “ECT2-interacting” and “ECT2-correlated” proteins. It was not a surprise that both of these proteins correlated with the progression of several types of human cancers [94,95,96,97]. Consequently, this interaction pathway could be a potential target for novel antitumor medications.

In continuation of our attempt to fully highlight the potentiality of ECT2 as a promising antitumor target, we explored its druggability for inhibition via small molecules. Small drug-like ligands are the cornerstone for drug discovery and development programs being broadly persued for targeting oncogenic proteins and their signaling pathways [98]. Such ligands harbor the ease of structural alterations while maintaining the optimized kinetic profiles, which allows their survival through the rigors of lead optimization and clinical development stages. Additionally, exhibiting drug-like properties favors the administration of small non-peptide ligands orally, the most convenient route [99]. To date, the pharmacological inhibition of RhoGEF is still elusive, yet several attempts have been introduced for encountering the aberrant nuclear oncogenic protein kinase C iota/RhoGEF/GTPase signaling cascade [100]. Clinical investigation of the FDA-approved small molecule drug, auranofin, as a promising protein kinase C iota inhibitor with antitumorogenic and antimetastatic activities, has been introduced [101,102]. However, concerns regarding drug-target selectivity have been raised and have represented great challenges against drug development [103]. In order to receive attention regarding another potential target, researchers have focused on inhibiting Rho GTPase proteins by targeting the surface grooves of Rac1. Small molecules identified from virtual screening approaches showed antiinvasive and antiproliferative activities on different cancerous cells, including malignant glioma brain neoplasms, aggressive breast carcinoma, and/or prostate cancerous cell lines [104,105,106]. Despite promising attempts, several other Rho GTPases considered less traditionally druggable have shown globular architectures with limited accessible surface grooves, which would hamper the scope of drug discovery/development efforts [107,108]. Such a drawback has caused research attention to focus on targeting RhoGEFs.

Notably, the RhoGEF DH-PH interface model represented a more druggable site for high-affinity chemical bindings, being concave, deep, and hydrophobically suitable for GTPase recognition and catalysis [108]. A successful approach was presented by Shang et al. targeting the DH-PH interface of LARG RhoGEF, showing SM1 as binding to the C-terminal DH-domain junction site with the PH region. SM1 managed to selectively hamper serum-driven RhoA activity and RhoA-associated signaling, as well as work synergistically with an inhibitor of Rho GTPase site-activation affecting mammalian breast cancerous cell lines. To our knowledge, identifying small ligand inhibitors for ECT2 is still elusive. Therefore, here, we reported for the first time molecular insights for targeting the ECT2–DH-PH interface site via small molecules in order to hamper the ECT2-mediated RhoGTPase-activated signaling pathway. The adopted ECT2 protein depicted a different topology compared with other reported RhoGEFs showing a significant autoinhibition ternary structure. The ECT2–DH-domain catalytic interface, which would be available for RhoA GTPase binding and recognition, was found totally sequestered via the PH domain, providing a mechanism for ECT2 autoinhibition [40]. Such architecture was recognized as similar to that depicted by son of sevenless (SOS), having its PH domain partially occlude the RhoGTPase binding site at the DH region [109]. Hydrophobic interactions primarily guide the PH-DH autoinhibition binding, being mediated via two anchoring thumb-like structures: (1) PH-domain β4-sheet tip Pro703 sidechain packing against DH-domain Leu574 and Leu575domain; (2) PH-domain αC-helix Cys765 sidechain into the pocket at DH-domain surface comprising Asn435, Arg564, and Arg568 residues. Mutagenesis studies at ECT2^P703D/C765K^ showed dramatic elevation of the RhoGEF activities beyond 10-fold increases. In this regard, the ability of small molecules to bind at the DH-PH interface site while being able to maintain the autoinhibition architecture of ECT2 would be considered promising to lack ECT2 at its inactive state.

Our study illustrated several small molecule ligands being suitable to bind at the DH-PH interface at high-chemical affinities, which was ensured through the molecular docking–coupled explicit dynamic approach. Docking of the investigated ligands at the DH-PH interface showed preferentiality for extended ligand structure, with the adaptation of hydrophobic functionalities such as aromatic/heterocyclic scaffolds. This was generally owed to the lipophilic topology of the ECT2 interface, which has also been seen with different RhoGEFs; such as Trio (PDB: 6D8Z), faciogenital dysplasia-5 (FGD5; PDB; 3MPX), p115 (PDB: 3P6A), FARP-2/1 (PDB: 4GZU and 4H6Y), and LARG (PDB: 1X86) [110,111,112]. Thermodynamic stability of several compounds was highlighted via monitored RMSD and RMSF trajectories, being optimum for almost all ligands except for SM7. Typically, altered conformational profiles and compromised stability are correlated with high protein RMSD values, whereas ligands with excellent pocket accommodation are related to steady and small-valued RMSD tones [113]. On the other hand, RMSF evaluates the residues’ dynamic behaviors (mobility/flexibility) by exploring the mean deviation of each protein amino acid in relation to its reference position across the simulated times. While being more accurate, RMSF can assess the fluctuations of a particular protein region from the average structure. This analysis tool would permit us to grasp the residue-wise dynamic behaviors at the protein’s binding pocket/vicinal loops in addition to pinpointing the key amino acids being significant for the ligand’s anchoring [114,115].

To our delight, molecular dynamics findings highlighted the significance of the preliminary docking residues for stabilizing ligands at the ECT2 site. Additionally, RMSF and conformational analysis highlighted the ability of the DH-PH site-confined simulated ligands, or even those drifted at the αC-helix of the PH domain, to keep the autoinhibition architecture. Finally, correspondence to the ECT2 interface nature predominance of van der Waal interactions was ensured through MM_PBSA free binding energy calculations. Molecular insights from ligand/ECT2 studies provided guidance for promising ligand structure modification and optimization. Improving the ligand’s inherited lipophilicity would boost chemical affinity towards the ECT2. Nevertheless, such an approach would increase the desolvation enthalpy needed to be compensated owing to highly ordered water molecules at the ligand/interface surfaces. In turn, providing balanced hydrophobic/polarity would be advantageous, yet increasing electrostatic potentiality could be associated with higher polar solvation penalties compromising binding and target affinity, since binding is a solvent displacement process. Therefore, a better lead optimization approach was suggested by introducing lipophilic functionalities with inherited ionizable characteristics that would provide a balanced deal for improving the ligand’s pharmacodynamic and kinetic/ADME profiles. Moreover, such polar decorating functional groups would satisfy the few polar residues (Gln567, Arg639, and Ser640) settled at the deep ECT2 interface, providing points for target selectivity. Examples of such moieties include the tetrazole ring and carboxylate bioisosteres.

## 5. Conclusions

In the current study, we employed a multi-omics analysis to assess the roles of ECT2 in tumor progression. ECT2 was found to be highly expressed in tumor tissues versus normal ones. Moreover, this overexpression predicted a progression in tumor stage and grade and a poor clinical outcome in a list of human tumors, where the genetic alterations in ECT2 also predicted poor patient survival. ECT2 also interfered with the infiltration of immune cells, where it allowed for the infiltration of immune suppressor cells. Due to these oncogenic roles, ECT2 could be selected as a target for antitumor therapies, where the current study employed a chemoinformatic approach to assess several inhibitors for ECT2. Future wet lab assessments are required to confirm the findings of the current study.

## Figures and Tables

**Table 1:** Dissected docking–binding interactions for small molecules at DH-PH catalytic interface of RhoGEF ECT2 and/or LARGE.

Small Molecules		Docking Binding Energy (Kcal.mol^−1^; RMSD Å)	H-Bond Interaction (Length Å/angle°)	Hydrophobic Interaction	π-Driven Interaction (Length Å)	van der Waal with Side Chain Carbons
Code-Target	Chemical Structure					
**SM1-ECT2**	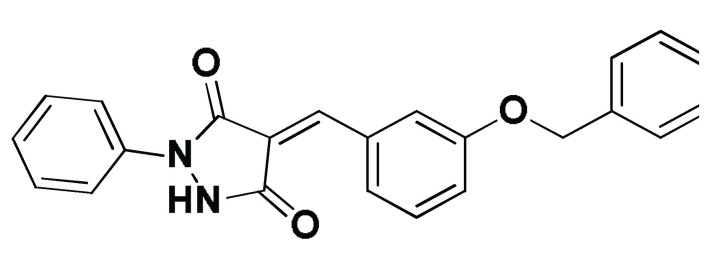	−5.824 (1.890)	Lys527; 2.5/121 Gln567; 2.4/159 Asp610; 2.9/140 Arg612; 3.0/126	Val520, Phe523, Val559, Ile563, Val566, Ala616, His638	Phe523; 5.1 Arg612; 3.2	Glu524 (Cβ,Cγ) Lys613 (Cγ,Cδ)
**SM2-ECT2**	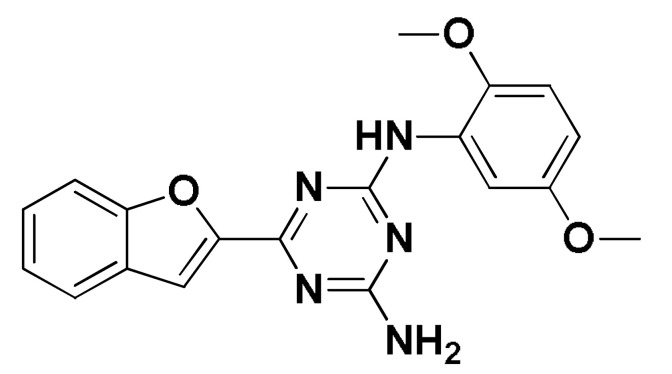	−6.369 (1.370)	Glu524; 2.6/124 Lys527; 1.9/158 Lys527; 2.4/129 Arg612; 2.7/125	Val520, Phe523, Ile563, Val566, Pro570, Ile607, Ala616, His638	Phe523; 5.0 Arg612; 3.1 Arg612; 2.0	Glu524 (Cβ,Cγ) Lys613 (Cβ,Cγ,Cδ)
**SM3-ECT2**	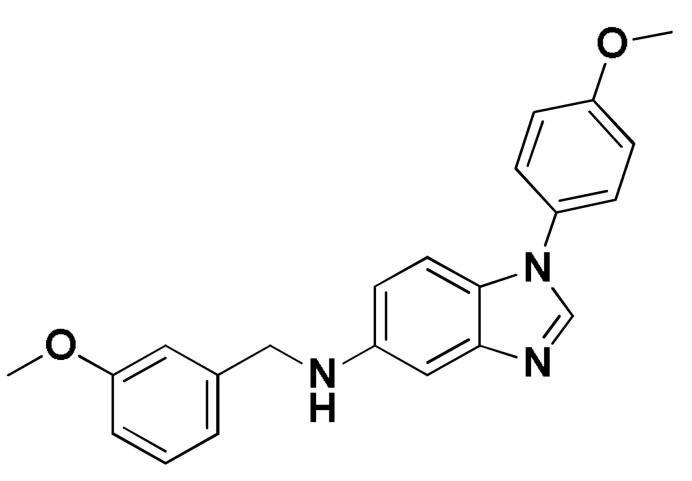	−6.061 (1.207)	Gln567; 3.2/133 Arg612; 2.6/130 Arg612; 2.4/128	Val520, Phe523, Ile563, Val566, Pro570, Ile607, Ala616, His638	Phe523; 4.9 Arg612; 3.1	Glu524 (Cβ,Cγ) Lys613 (Cγ,Cδ)
**SM4-ECT2**	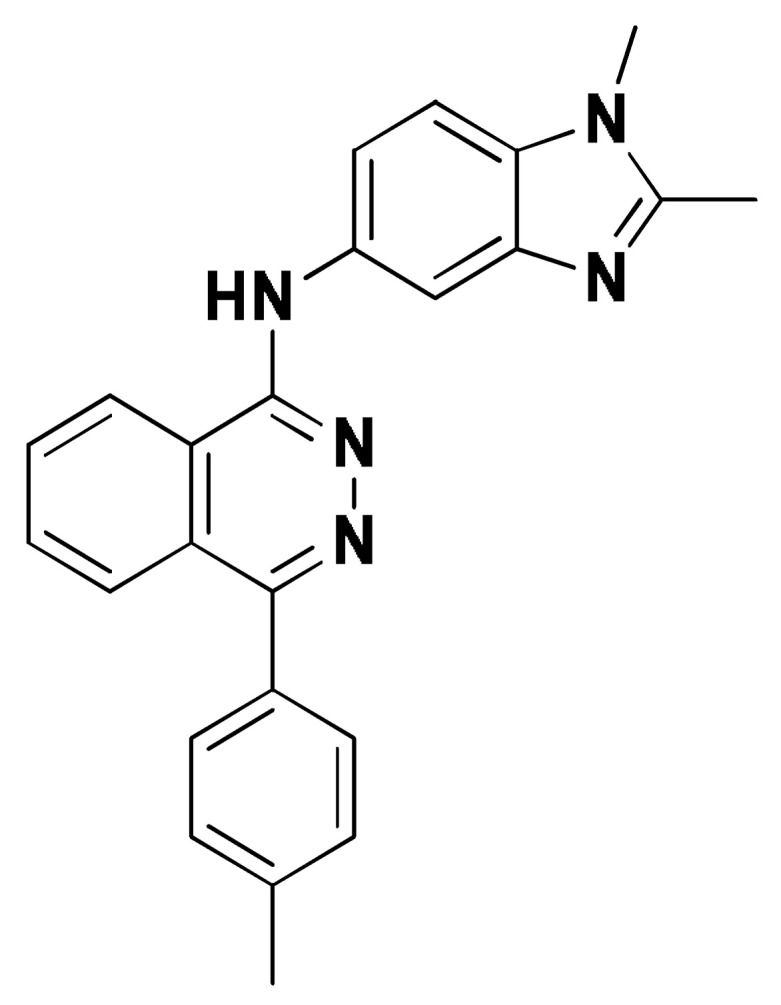	−5.855 (1.078)	Gln567; 3.2/133 Arg612; 2.7/121 Arg612; 2.3/131	Phe523, Val559, Ile563, Val566, Ala616, His638	Phe523; 5.0 Gln567; 5.1 Arg612; 3.1 Arg612; 3.0	Glu524 (Cβ,Cγ) Lys613 (Cβ,Cγ,Cδ)
**SM5-ECT2**	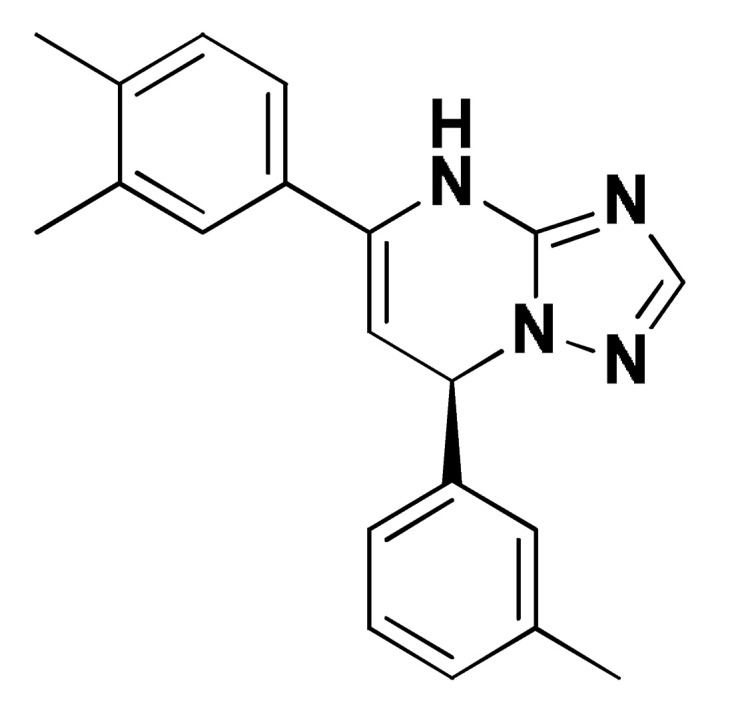	−4.972 (1.623)	Arg612; 1.3/143 Arg612; 3.2/126	Val520, Phe523, Ile563, Val566, Pro570, Ile607, Ala616, His638	Gln567; 3.7 Lys527; 3.0 Arg612; 2.7	Glu524 (Cγ) Lys613 (Cγ,Cδ)
**SM6-ECT2**	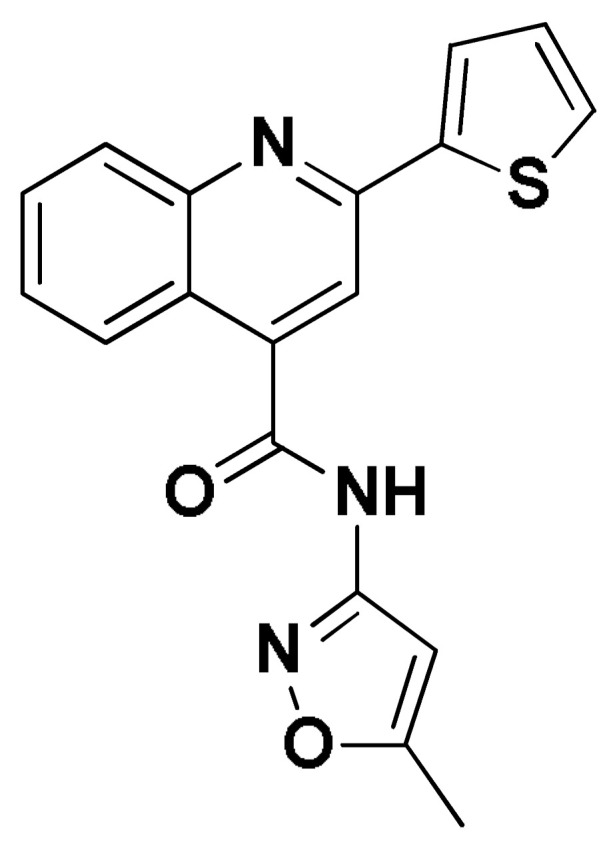	−4.853 (1.569)	Arg612; 2.5/131 Arg612; 2.7/129	Phe523, Val559, Ile563, Ala616, His638, His759	Phe523; 4.8 Glu567; 3.9 Arg612; 2.3	Glu524 (Cβ,Cγ) Lys613 (Cγ,Cδ)
**SM7-ECT2**	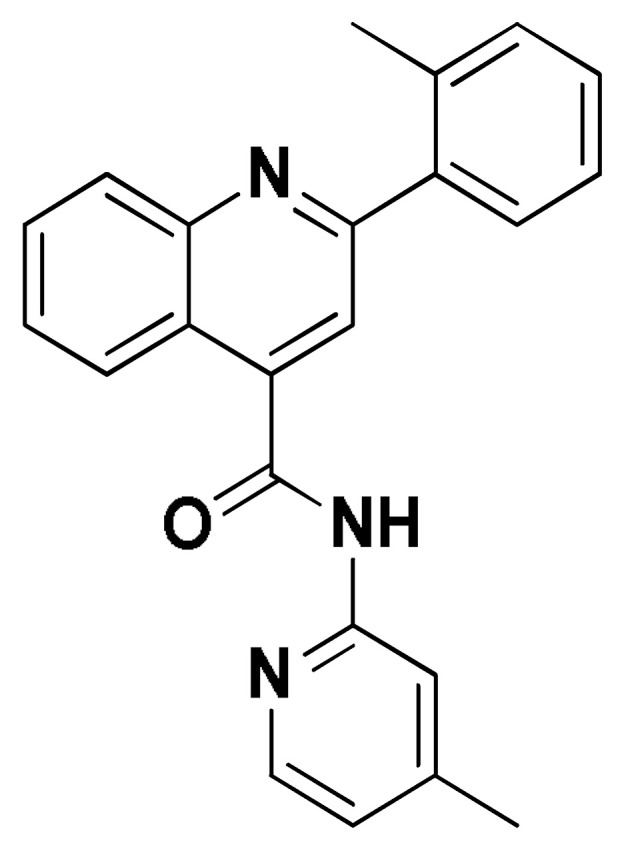	−5.469 (1.012)	Gln567; 2.4/159 Asp610; 2.9/140 Arg612; 3.0/120 Lys527; 2.5/126	Val520, Phe523, Val559, Ile563, Val566, Ala616, His638	Phe523; 4.8 Lys527; 4.7 Arg612; 3.2	Glu524 (Cβ,Cγ) Lys613 (Cβ,Cγ)
**SM1-LARG**	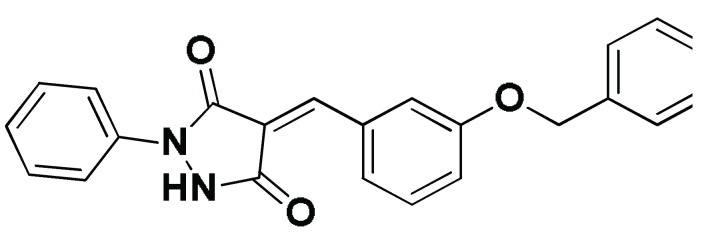	−5.534 (1.065)	Pro892; 3.2/146 Arg986; 2.5/143 Arg986; 2.1/159	Cys888, Phe892, Leu895, Met934, Leu937, Leu971, Val974, Val978	Phe893; 4.9	Glu896 (Cβ,Cγ) Asn975 (Cβ) Gln985 (Cβ,Cγ)

**Table 2:** Total free binding energy (ΔG ± SE) for ligand/RhoGEF systems via MM_PBSA approach.

Ligand/RhoGEF	Energy (kJ/mol ± SE)				
	ΔG_vdW_	ΔG_electrostatic_	ΔG_Solvation_	ΔG_SASA_	ΔG_Total_
**SM1-ECT2**	−115.21 ± 8.66	−7.83 ± 14.77	65.68 ± 7.55	−13.75 ± 2.15	−71.11 ± 9.84
**SM2-ECT2**	−114.20 ± 32.03	−36.58 ± 37.89	103.21 ± 30.75	−14.69 ± 3.95	−62.26 ± 21.79
**SM3-ECT2**	−103.52 ± 9.21	−30.26 ± 15.56	103.99 ± 32.83	−12.70 ± 2.44	−42.49 ± 14.68
**SM4-ECT2**	−133.56 ± 19.32	−35.22 ± 23.48	147.80 ± 30.33	−18.34 ± 1.70	−39.32 ± 24.11
**SM5-ECT2**	−116.85 ± 19.44	−5.20 ± 8.63	76.88 ± 31.92	−14.64 ± 2.23	−59.81 ± 17.65
**SM6-ECT2**	−135.18 ± 5.64	−36.59 ± 34.42	138.11 ± 48.95	−16.89 ± 0.75	−50.55 ± 16.45
**SM7-ECT2**	−58.45 ± 53.16	−5.80 ± 14.24	60.72 ± 43.58	−7.91 ± 7.26	−11.44 ± 69.52
**SM1-LARG**	−121.79 ± 10.85	−41.63 ± 16.39	109.01 ± 17.33	−16.09 ± 0.50	−70.50 ± 10.25

## Data Availability

The data presented in this study are available on request from the corresponding author.

## Supplementary Materials

The following supporting information can be downloaded at: https://www.mdpi.com/article/10.3390/biology12040613/s1, Figure S1: Tumors that showed a non-significant difference or elevated ECT2 expression levels in normal tissue versus the cancerous one through analysis on the GEPIA2 database; Figure S2: Tumors that experienced a non-significant difference in ECT2 protein level in cancerous tissues and normal ones; Figure S3: (A) The correlation between ECT2 expression level and infiltration of NKT cells. (B) Scatter plots that demonstrate the correlation between the expression of ECT2 and the infiltration level of NKT cells; Table S1: The abbreviations and the full name of analyzed tumors in the current study.
