# Supplementary material for: Integration of Chemoinformatics and Multi-Omics Analysis Defines ECT2 as a Potential Target for Cancer Drug Therapy
Source: Biology (Basel). 2023 Apr 18;12(4):613. doi: 10.3390/biology12040613 (PMC10135641; doi:10.3390/biology12040613)
Supplement: Supplementary file 1 [file biology-12-00613-s001.zip › biology-2262811-supplementary.pdf]

Integration of Chemoinformatics and Multi-Omics Analysis Defines ECT2 as a Potential Target for Cancer Drug Therapy

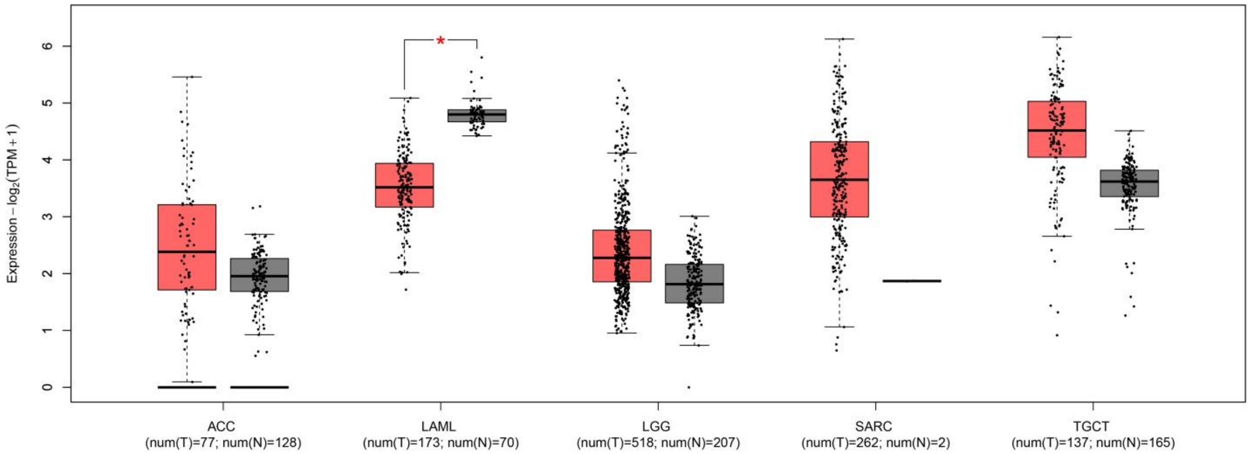

Supplementary Figure S1. Tumors that showed a non-significant difference or elevated ECT2 expression levels in normal tissue versus the cancerous one through analysis on the GEPIA2 database.

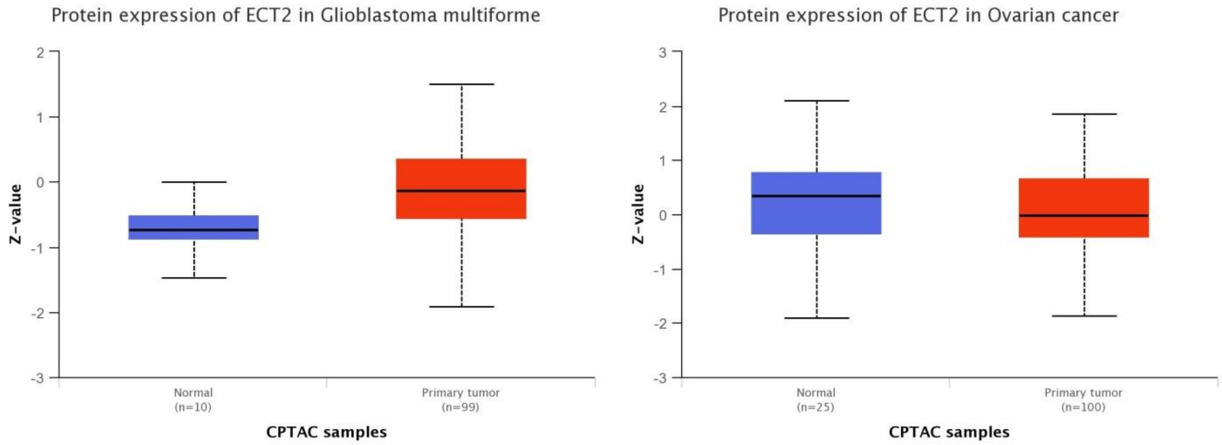

Supplementary Figure S2. Tumors that experienced a non-significant difference in ECT2 protein level in cancerous tissues and normal ones.

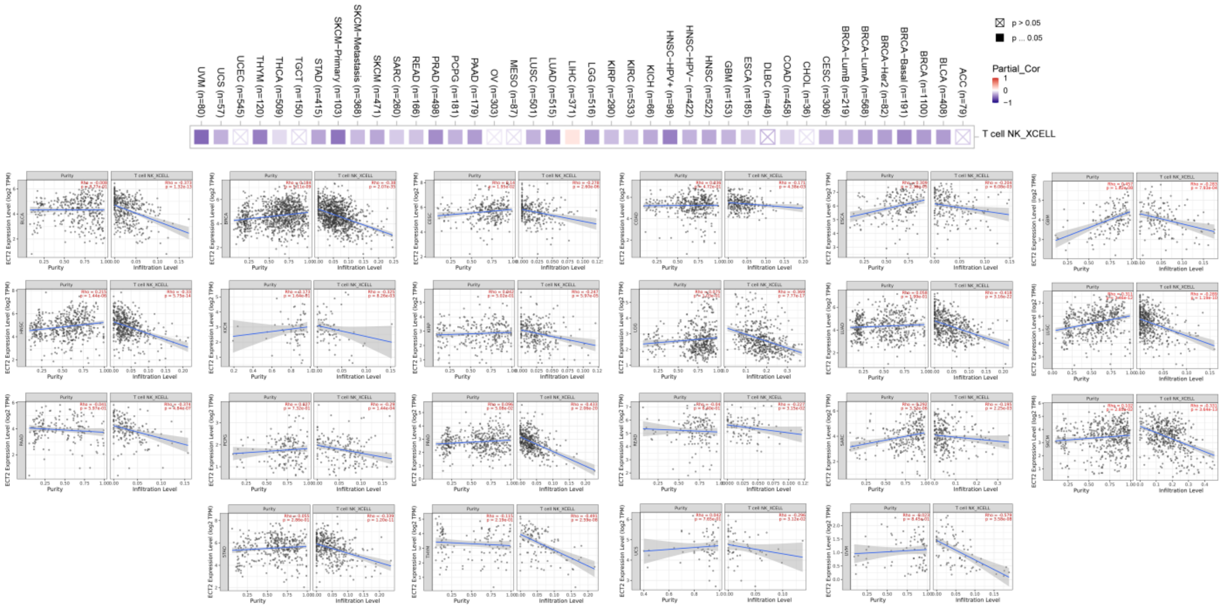

Supplementary Figure S3. (**A**) The correlation between ECT2 expression level and infiltration of NKT cells. (**B**) Scatter plots that demonstrate the correlation between the expression of ECT2 and the infiltration level of NKT cells.

Supplementary Table S1. The abbreviations and the full name of analyzed tumors in the current study.

Abbrviation	Tumor Name
ACC	Adrenocortical carcinoma
BLCA	Bladder Urothelial Carcinoma
BRCA	Breast invasive carcinoma
CESC	Cervical squamous cell carcinoma and endocervical adenocarcinoma
CHOL	Cholangiocarcinoma
COAD	Colon adenocarcinoma
DLBC	Lymphoid Neoplasm Diffuse Large B-cell Lymphoma

ESCA	Esophageal carcinoma
GBM	Glioblastoma multiforme
HNSC	Head and Neck squamous cell carcinoma
KICH	Kidney Chromophobe
KIRC	Kidney renal clear cell carcinoma
KIRP	Kidney renal papillary cell carcinoma
LAML	Acute Myeloid Leukemia
LGG	Brain Lower Grade Glioma
LIHC	Liver hepatocellular carcinoma
LUAD	Lung adenocarcinoma
LUSC	Lung squamous cell carcinoma
MESO	Mesothelioma
OV	Ovarian serous cystadenocarcinoma
PAAD	Pancreatic adenocarcinoma
PCPG	Pheochromocytoma and Paraganglioma
PRAD	Prostate adenocarcinoma
READ	Rectum adenocarcinoma
SARC	Sarcoma
SKCM	Skin Cutaneous Melanoma
STAD	Stomach adenocarcinoma
TGCT	Testicular Germ Cell Tumors
THCA	Thyroid carcinoma
THYM	Thymoma
UCEC	Uterine Corpus Endometrial Carcinoma
UCS	Uterine Carcinosarcoma
UVM	Uveal Melanoma
